# Employing Piezoelectric Mg^2+^‐Doped Hydroxyapatite to Target Death Receptor‐Mediated Necroptosis: A Strategy for Amplifying Immune Activation

**DOI:** 10.1002/advs.202307130

**Published:** 2024-01-22

**Authors:** Jiani Yang, Yaqian Du, Yuanfei Yao, Yuanyu Liao, Bojun Wang, Xuefan Yu, Kaikun Yuan, Yanqiao Zhang, Fei He, Piaoping Yang

**Affiliations:** ^1^ Department of Gastrointestinal Medical Oncology Harbin Medical University Cancer Hospital Harbin 150001 P. R. China; ^2^ Key Laboratory of Tumor Immunology in Heilongjiang Harbin Medical University Cancer Hospital Harbin 150080 China; ^3^ Key Laboratory of Superlight Materials and Surface Technology Ministry of Education College of Materials Science and Chemical Engineering Harbin Engineering University Harbin 150001 P. R. China; ^4^ Department of Neurosurgery First Affiliated Hospital of Harbin Medical University Harbin 150001 P. R. China

**Keywords:** DR5, hydroxyapatite, immunotherapy, necroptosis, piezoelectric catalysis

## Abstract

Although immunogenic cell death (ICD) inducers evidently enhance the effectiveness of immunotherapy, their potential is increasingly restricted by the development of apoptosis resistance in tumor cells, poor immunogenicity, and low T‐cell immune responsiveness. In this study, for the first time, piezoelectrically catalyzed Mg^2+^‐doped hydroxyapatite (Mg‐HAP) nanoparticles, which are coated with a mesoporous silica layer and loaded with ONC201 as an agonist to specifically target the death receptor DR5 on tumor cells, ultimately developing an Mg‐HAP@MS/ONC201 nanoparticle (MHMO NP) system, are engineered. Owing to its excellent piezoelectric properties, MHMO facilitates the release of a significant amount of reactive oxygen species and Ca^2+^ within tumor cells, effectively promoting the upregulation of DR5 expression and inducing tumor cell necroptosis to ultimately overcome apoptosis resistance. Concurrently, Mg^2+^ released in the tumor microenvironment promotes CD8^+^ T receptor activation in response to the antitumor immune reaction induced by ICD. Using RNA‐seq analysis, it is elucidated that MHMO can activate the NF‐κB pathway under piezoelectric catalysis, thus inducing M1‐type macrophage polarization. In summary, a dual‐targeting therapy system that targets both tumor cells and the tumor microenvironment under piezoelectric catalysis is designed. This system holds substantial potential for advancements in tumor immunotherapy.

## Graphical abstract

The MHMO NPs exhibit superior piezoelectric properties. The released ROS, Ca^2+^, and ONC201 effectively induce DR5 expression, activating necroptosis to overcome apoptosis resistance and inducing DC maturation and M1 polarization. Additionally, the released Mg^2+^ enhances T‐cell receptor activation to better respond to ICD. This strategy effectively achieves the dual targeting of both tumor cells and the microenvironment.

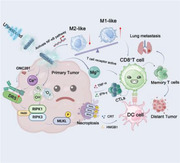

## 1 Introduction

Colorectal cancer (CRC), ranking as the third most prevalent malignancy globally, exhibits a notably high mortality rate.^[^ ^1^ ^]^ With advancements in the exploration of interactions between cancer cells and the immune microenvironment, the development of immune checkpoint inhibitors (ICIs) has markedly enhanced the overall survival of patients with cancer. However, ICIs have demonstrated limited clinical efficacy when used in patients with CRC.^[^ ^2^ ^]^ Although microsatellite instability (MSI) is considered a predictive biomarker for effective immunotherapy in colorectal cancer, the proportion of patients with high MSI (MSI‐H) is relatively small. In contrast, most patients have microsatellite‐stable (MSS) CRC, and therefore, do not benefit from ICIs.^[^ ^3^ ^]^ Consequently, the challenge lies in enhancing tumor immunogenicity, increasing immune infiltration, and enhancing the response to ICIs in these patients.^[^ ^4^ ^]^ Targeting death receptors, such as DR5, can induce tumor cell death, leading to immunogenic cell death (ICD).^[^ ^5^ ^]^ However, its effectiveness is often reduced by apoptosis resistance and lethal inflammatory damage caused by systemic administration. Enhancing DR5 expression and exploiting alternative pathways to overcome apoptosis resistance have become focal points for the treatment of CRC. ONC201, an agonist of the ligand TRAIL, facilitates the interaction between TRAIL and its receptor DR5, thereby driving tumor cell death. However, its short serum half‐life and poor stability limit its clinical applications. In addition, an increase in intracellular reactive oxygen species (ROS) and Ca^2+^ levels induces the upregulation of DR5, thereby increasing necroptosis—the second‐line defense mechanism against tumor progression in cases of apoptosis resistance.^[^ ^6^ ^]^ Simultaneously, necroptosis induced by ROS and upregulated Ca^2+^ within cells elicits increased inflammation compared to apoptosis, effectively inducing ICD and transforming “cold” tumors into “hot” ones.^[^ ^7^ ^]^ In addition to reducing immunogenicity through the necroptosis of tumor cells, Mg^2+^ release enhances the immune response of T cells.^[^ ^8^ ^]^ Recently, the emergence of nanomedicine in tumor therapy has attracted considerable attention^[^ ^9^ ^]^ owing to its superior drug delivery targetability, efficient ROS generation, and ion release.^[^ ^10^ ^]^

**Scheme 1:**
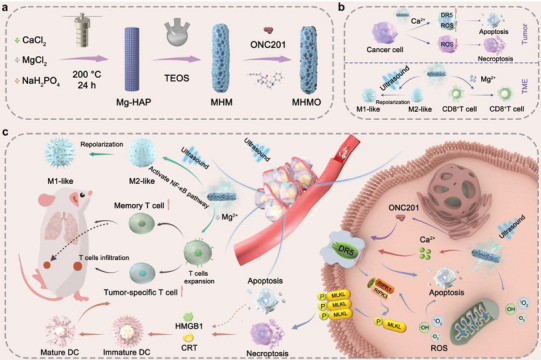
a) A diagrammatic illustration of the MHMO synthesis process. b) Dual regulation of MHMO in tumor cells and the tumor microenvironment under piezoelectric stimulation. c) MHMO + ultrasound (US) induces death receptor DR5 upregulation, thereby promoting tumor cell death and triggering a cascade activation of various immune cells in the immune microenvironment.

Piezoelectric‐catalyzed therapy represents an innovative approach to tumor treatment.^[^ ^11^ ^]^ Under the influence of externally applied mechanical stress, piezoelectric catalysts generate an electric field that induces the separation and surface migration of charge carriers, culminating in the production of reactive free radicals.^[^ ^12^ ^]^ Exploiting the piezoelectric effects induced by mechanical energy to inhibit tumor growth represents a promising strategy in oncological research.^[^ ^13^ ^]^ Hydroxyapatite (Ca_10_(PO_4_)_6_(OH)_2_; HAP) is the predominant mineral in bones and teeth and has drawn substantial interest as a biomimetic material for diverse applications owing to its unique structure and inherent properties.^[^ ^14^ ^]^ Its excellent biocompatibility and chemical stability facilitate its wide application in areas such as bone grafts, prosthetic coatings,^[^ ^15^ ^]^ drug delivery systems,^[^ ^16^ ^]^ and multiphase catalysis.^[^ ^17^ ^]^ However, its potential as a novel piezoelectric material in the field of biomaterials, particularly in piezoelectric‐catalyzed therapies, remains unexplored. HAP exhibits a crystalline structure that conforms to a hexagonal system, specifically, the P63/m space group. This particular characteristic distinguishes it from classical piezoelectric crystal point groups. Its piezoelectric traits stem from the ferroelectric arrangement of ·OH ions in the [001] direction. Previous studies have indicated that Mg^2+^ can exert specific cytotoxicity and eliminate tumor cells by modulating the active conformation of the LFA‐1 receptor on CD8^+^ T cells. Likewise, elevated serum magnesium levels have been correlated with prolonged overall survival in patients.^[^ ^8^ ^]^ Consequently, we incorporated Mg^2+^ into HAP to achieve immune reprogramming in the tumor microenvironment (**Scheme** **1**a). Simultaneously, to enhance the targeting and activation of the death receptor DR5, we incorporated its ligand agonist ONC201 to obtain an Mg‐HAP@MS/ONC201 nanoparticle (MHMO NP) system.

The ultrasonication‐induced polarization of MHMO facilitates sono‐excited charge separation, and the diverging electrons and holes at opposing interfaces initiate redox reactions, consequently generating cytotoxic ROS. The Ca^2+^ released can synergize with ROS to effectively induce DR5 upregulation, thereby triggering necroptosis and inducing ICD (Scheme 1b). Additionally, RNA‐Seq data suggests that the abundant inflammatory responses induced by necroptosis stimulate NF‐κB pathway activation, leading to M1‐type macrophage polarization (Scheme 1c). Meanwhile, Mg^2+^ release prompts the activation of the T‐cell receptor signaling pathway. Consequently, we have devised a dual‐target therapy that leverages Ca^2+^, ROS, and ONC201 to induce necroptotic apoptosis in tumor cells, enhance ICD, and utilize Mg^2+^ release for T‐cell receptor activation. These various components play crucial roles in antitumor therapy, ultimately enhancing the response to ICD and achieving the dual targeting of tumor cells and the immune microenvironment.

## 2 Results and Discussion

### 2.1 Synthesis and Characterization of MHMO

**Figure 1:**
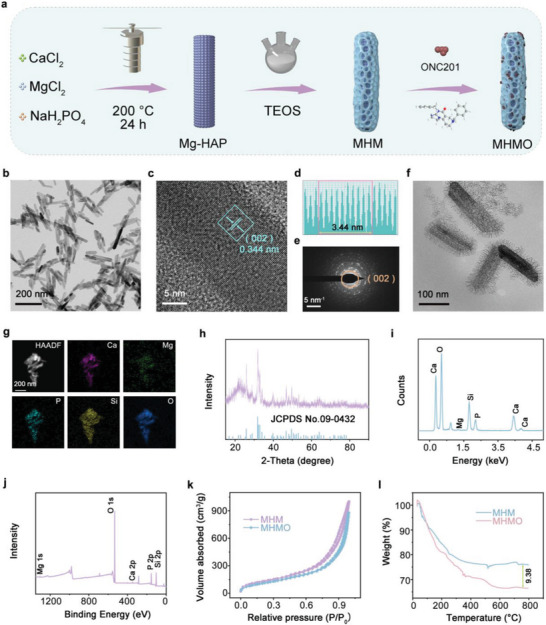
Synthesis and characterization of Mg‐HAP@MS/ONC201 (MHMO). a) Synthesis scheme of MHMO NPs. b) TEM and c, d) HRTEM images of Mg‐HAP NPs. e) SAED pattern of MHM NPs. f) TEM image of MHM NPs. g) Elemental mapping and HAADF‐STEM imaging of MHMO NPs. h) X‐ray diffraction (XRD) pattern of MHMO NPs. i) Energy‐dispersive X‐ray spectroscopy (EDS) spectrum of MHMO NPs. j) X‐ray photoelectron spectroscopy (XPS) spectrum of the MHMO NPs. k) The N_2_ adsorption‐desorption isotherm curves of MHM and MHMO NPs. l) Thermogravimetric analysis of MHM and MHMO NPs.

The detailed synthesis of the MHMO NPs is shown in **Figure** **1**a. By adjusting the Ca: Mg ratio for system optimization, we observed that a ratio of 3.9:0.1 (as shown in Figure S1a, Supporting Information) led to diminished T‐cell receptor activation owing to low Mg. Conversely, a ratio of 3.7:0.3 (depicted in Figure S1b, Supporting Information) led to structural collapse, deviating from the design objective. However, the ideal ratio of 3.8:0.2 maintained morphology and effectively activated T‐cell receptors. The hydrothermally synthesized Mg‐HAP NPs exhibited a uniform rod‐shaped morphology, with an average length of ≈99.9 ± 10.8 nm (Figure 1b). As shown in Figure 1c–e, the lattice spacing of 0.344 nm corresponded to the crystal planes indexed as (002) in the HAP structure. The Mg‐HAP NPs underwent a surface‐coating procedure using mesoporous silica in an oil‐water biphasic reaction environment. In this context, cyclohexane served as the hydrophilic solvent in the upper tetraethyl orthosilicate (TEOS) oil phase, whereas the cationic cetyltrimethylammonium chloride (CTAC) template formed the lower aqueous phase. Finally, ONC201 was loaded into the pores of silica by mechanical stirring, forming the MHMO NPs. Following the modification process, the mean dimensions of the MHMO NPs expanded to 65.6 ± 9.2 nm in width and 110.6 ± 18.6 nm in length (Figure 1f), and the image revealed a distinctive dendritic pore structure. The homogeneous distributions of Ca, Mg, Si, P, and O in the MHMO NPs were confirmed by elemental mapping (Figure 1g), illustrating the successful formation of the MHMO NPs.

Additionally, an X‐ray diffraction (XRD) analysis of the MHMO NPs was conducted (Figure 1h). The characteristic peaks belonging to hexagonal hydroxyapatite (JCPDS No. 09–0432) were evident, indicating that a small amount of Mg^2+^ doping had a minimal effect on the crystal structure of HPA. Notably, the wide peak at ≈22° can be attributed to amorphous silica, indicating successful coating with silica. Furthermore, energy‐dispersive X‐ray spectroscopy (EDS) analysis of the MHMO revealed the coexistence of Ca, Mg, Si, P, and O signals (Figure 1i). The presence of Ca, Mg, Si, P, and O within the MHMO NPs was further validated by X‐ray photoelectron spectroscopy (XPS) spectra (Figure 1j).

Figure S2 (Supporting Information) shows the diverse size distributions of MHMO NPs after incubation in phosphate‐buffered saline (PBS), simulated body fluid (SBF), saline solution, and RPMI‐1640 medium (1640) for 12, 24, 36, and 48 h periods. Figure 1k and Figure S3 (Supporting Information) illustrate the isotherm curves of N_2_ adsorption‐desorption and the corresponding pore size distributions of the MHM and MHMO NPs. Compared to MHM (477 m^2^ g^−1^), MHMO (414 m^2^ g^−1^) exhibited a reduced specific surface area and a decrease in the corresponding pore size, further suggesting that ONC201 was loaded into the pores of the mesoporous silica. Figure S4 (Supporting Information) illustrates the *zeta* potential dynamics of materials synthesized at different stages. Owing to the negative charge of ONC201, the negative potential value of the MHMO NPs increased, further indicating the successful integration of ONC201. Figure 1l presents the results of the thermogravimetric analysis (TGA) conducted on various samples. The weight loss of the MHMO NPs was 9.38% higher than that of the MHM NPs, owing to the thermolysis of the loaded ONC201. The loading capacity (LC) and encapsulation efficiency (EE) values of ONC201 were nearly 10.3% and 19.1%, respectively.

A linear relationship was observed between the absorbance of free ONC201 at 388 nm and the drug concentration, as illustrated in Figure S5a (Supporting Information). Moreover, we evaluated the in vitro release behavior of ONC201 in MHMO NPs at pH 6.5, pH 7.4, and pH 6.5 + US (Figure S5b, Supporting Information). An acidic environment and US exposure accelerated the release rate of ONC201 from the MHMO. This observation implied that piezoelectrically modulated drug release from MHMO could enhance localized ONC201 dispersal, thereby minimizing the adverse effects of systemic inflammation resulting from ONC201 leakage into the bloodstream.

### 2.2 In Vitro Assessment of the Piezoelectric Catalytic Activities of MHMO

**Figure 2:**
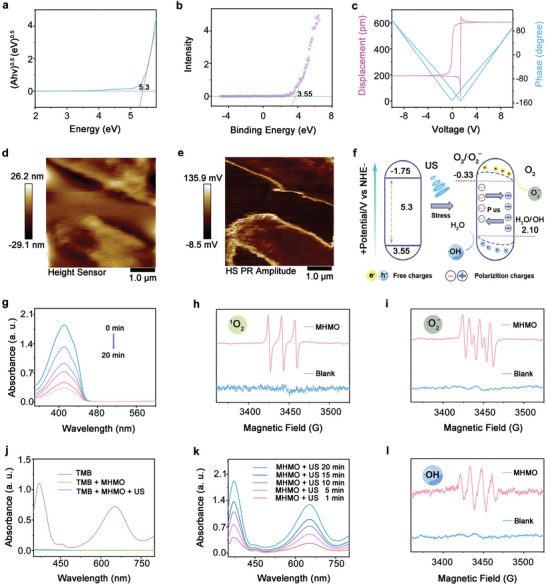
Piezoelectric performance evaluation and ROS measurement. a) Bandgap energies and b) XPS analysis showing the VB of MHMO. c) Phase and displacement response represented by piezoresponse force microscopy (PFM) analysis of the MHMO NPs. d) PFM morphology of the MHMO NPs. e) Amplitude diagram of the MHMO NPs. f) Schematic representation of the intrinsic band of the MHMO NPs and the tilted energy band that enhances the formation of ·O_2_^−^ and ·OH in the presence of piezoelectric fields induced by US exposure. g) Assessment of ·O_2_^−^ and ^1^O_2_ generation under US irradiation of MHMO NPs, with 1,3‐diphenylisobenzofuran (DPBF) and 2,2,6,6‐tetramethylipiperidine (TEMP) used as  probe. h) Electron spin resonance (ESR) spectral analysis of ^1^O_2_ captured by TEMP. i) ESR spectral analysis of ·O_2_^−^ captured by DMPO. j) Evaluation of oxTMB UV–vis spectral responses across various conditions. k) The time‐dependency of TMB oxidation is attributable to ·OH generated from MHMO NPs. l) ESR spectral analysis of ·OH captured by DMPO.

For a deeper understanding of the piezoelectric attributes of the MHMO NPs, their light‐absorption capacity was assessed using ultraviolet‐visible (UV–vis) diffuse reflectance spectroscopy (Figure S6, Supporting Information). Moreover, the corresponding bandgap energies were inferred from the graph of (αhv)^0.5^ versus light energy uptake (**Figure** **2**a), with the MHMO NPs having a

calculated bandgap of 5.3 eV. Evaluation of the conduction band (CB) and valence band (VB) positions of sonosensitizers is essential. The VB of MHMO was investigated using XPS (Figure 2b) and was revealed to be +3.55 eV. The CB position of the samples was determined using the equation *E*_CB_ = *E*_VB_ – *E*_g_. Consequently, the CB of the MHMO NPs was estimated to be −1.75 V. The piezoelectric characteristics of the MHMO NPs were validated through piezoresponse force microscopy (PFM), as shown in Figure 2c–e. Figure S7 (Supporting Information) shows the PFM amplitude, phase images, and corresponding curves of the MHMO NPs. The *d*_33_ of the MHMO NPs was 58 pm/V, which is higher than the reported piezoelectric coefficients of HAPs and even higher than those of most other lead‐free perovskites.

The phase image of the MHMO NPs reveals a distinct phase variance, with the image contrast inversely correlated with the amplitude signal. An amplitude–voltage hysteresis curve resembling a butterfly loop was observed upon applying an alternating current voltage to the NS. This characteristic is commonly observed in ferroelectric materials.^[^ ^15^, ^18^ ^]^ As indicated by the phase diagram, an average phase contrast of ≈180° was observed, confirming the piezoelectric attributes of the MHMO NPs. Figure S8 (Supporting Information) presents the results of US current testing conducted on the MHMO NPs under continuous US exposure. A steady response to the US current was observed across five repetitive cycles of MHMO NPs. The observed high stability and strong electrical current signal suggested that the MHMO NPs could promote the migration and separation of charge carriers under US exposure.

The pressure generation sequence is illustrated in Figure S9 (Supporting Information). Upon propagation of US waves through the liquid medium, the acoustic pressure manifested as a wave pattern, leading to the formation of several cavitation bubbles. Subsequently, the bubbles evolved and ultimately collapsed. The bubble implosion liberated substantial energy, which manifested as light emission, elevated temperature, and high pressure. The ensuing impact on the adjacent MHMO can reach 1 × 10^8^ Pa, suggesting the periodic generation of a piezoelectric‐induced potential.^[^ ^18^ ^]^ To substantiate the interrelation between piezoelectric properties and sonodynamic behavior, it is crucial to evaluate the band alignment. When subjected to ultrasonic stress, the MHMO NPs exhibited a distinct migration pattern for positive and negative charges, which was consistent with internal polarization. Concurrently, internal polarization led to band bending, which was characterized by an increase in the band energy at the CB side and a reduction at the VB side. Band bending diminished the distance between the band edge and the redox potentials of O_2_/·O_2_^–^ (−0.33 V) and H_2_O/·OH (+2.10 V). Consequently, the facilitation of e^−^–h^+^ separation occurred and catalyzed the energetic reactions between e^−^/h^+^ and O_2_/H_2_O, culminating in ROS generation (Figure 2f).

To explore the piezoelectric catalytic activity of MHMO NPs, 1,3‐diphenylisobenzofuran (DPBF) was employed as a detector for the superoxide anion radical (·O_2_^−^) and singlet oxygen (^1^O_2_) following US irradiation.^[^ ^19^ ^]^ As illustrated in Figure 2g, in the MHMO‐treated group, the peak gradually weakened with prolonged US irradiation time, thereby validating piezoelectric‐triggered ROS production. Furthermore, the electron spin resonance (ESR) spectrum (Figure 2h,i) provided further confirmation of ^1^O_2_ and ·O_2_^−^ generation. Within the methanol system, ^1^O_2_ was trapped by 2,2,6,6‐tetramethylpiperidine (TEMP), while ·O_2_^−^ was trapped by 5,5‐dimethyl‐1‐pyrroline N‐oxide (DMPO). Thereafter, 3,3,5,5‐tetramethyl‐benzidine (TMB) was employed as a probe to monitor changes in the absorbance at 652 nm, enabling the quantification of ·OH production during the course of piezoelectric catalysis (Figure 2j,k). As shown in Figure 2j, the minimal absorbance observed for both TMB alone and the TMB + MHMO combination suggested the absence of an oxidation reaction. The two characteristic absorbance peaks at 370 and 652 nm could be attributed to oxidized TMB (oxTMB), implying that ·OH was generated via piezoelectric catalysis under US. Moreover, as the US exposure time increased, the peak demonstrated a gradual increase, indicating the progressive generation of additional ·OH. This was further validated by the characteristic peak intensity of the DMPO‐·OH adducts observed in the ESR spectra (Figure 2l).

The release of Ca^2+^ and Mg^2+^ from MHMO under different conditions was monitored by ICP‐OES (Figure S10, Supporting Information). The results showed that under neutral conditions (pH 7.4), there was almost no release of Ca^2+^ and Mg^2+^, and the MHMO structure remained relatively stable. Under weakly acidic conditions (pH 6.5), a large proportion of Ca^2+^ and Mg^2+^ was released, indicating that MHMO underwent acidic degradation, leading to structural damage and the release of Ca^2+^ and Mg^2+^. After the application of US, the release capability was further amplified, demonstrating a significant auxiliary therapeutic effect.

### 2.3 In Vitro Antitumor Activity of MHMO

Previous investigations have revealed that elevated intracellular ROS and Ca^2^⁺ levels can significantly upregulate DR5 expression, thereby triggering the activation of cell death pathways.^[^ ^6^ ^]^ In apoptosis‐insensitive tumor cells, necroptosis serves as a backup cell death pathway, serving as a compensatory mechanism to overcome defective apoptotic processes.^[^ ^20^ ^]^

**Figure 3:**
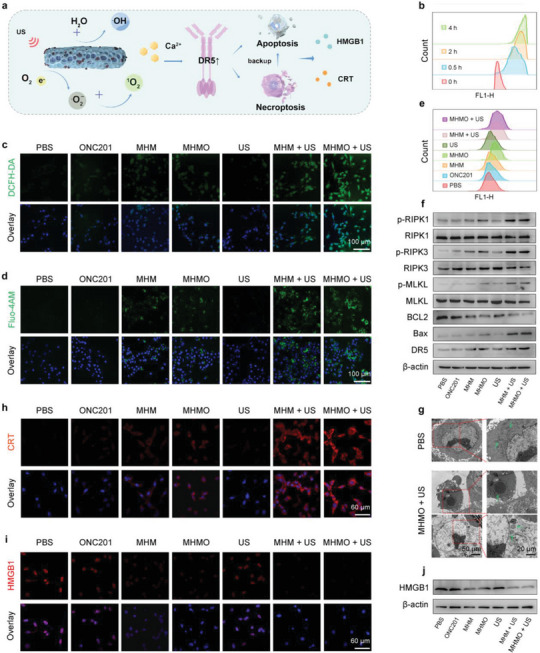
Apoptosis, necroptosis, and immunogenic cell death (ICD) in vitro through the upregulation of death receptor DR5. a) The schematic diagram illustrates the release of ROS and Ca^2+^ by MHMO under piezoelectric stimulation, leading to DR5 upregulation, promoting apoptosis, with necroptosis serving as a backup mechanism, and ultimately inducing ICD. b) Flow cytometry visualization of MHMO uptake at various time points. c) Confocal microscopy demonstrates ROS production in different treatment groups. The scale bar represents 100 µm. D) Fluo‐4 Acetoxymethyl Ester (Fluo‐4AM) staining reveals intracellular Ca^2+^ levels in different treatment groups. The scale bar represents 100 µm. e) Following treatment, cells were stained with JC‐1 and flow cytometry was used to detect JC‐1 monomers in each treatment group. f) Protein extraction from post‐treatment CT26 cells was performed, and the expression levels of necroptosis markers (p‐RIPK1, RIPK1, p‐RIPK3, RIPK3, p‐MLKL, and MLKL), apoptosis markers (Bax and Bcl2), and death receptor DR5 were detected by western blotting. g) Bio‐Transmission Electron Microscopy (Bio‐TEM) images showing morphological changes in CT26 cells before and after MHMO + US treatment. The images from the top to the bottom represent normal, apoptosis, and necroptosis, respectively. Arrows indicate normal mitochondria (normal), nuclear alterations (apoptosis), translucent cytoplasm, and organelle swelling (necroptosis). h, i) Confocal microscopy demonstrates the expression levels of ICD markers (HMGB1 and CRT) in different treatment groups. The scale bar represents 60 µm. j) Western blotting depicting HMGB1 expression in different treatment groups.

**Figure** **3**a illustrates that MHMO induces the release of ROS and Ca^2^⁺ upon US exposure, facilitating the activation of apoptosis and necroptosis. The biosafety of the prepared MHMO was evaluated via hemolysis assays (Figure S11a, Supporting Information). No hemolysis was observed across the evaluated concentrations of MHMO compared to the control group, which was consistent with the visual observations (Figure S11b, Supporting Information).

In this study, we co‐cultured 3T3 cells (a murine embryonic fibroblast cell line) with varying concentrations of MHMO to determine whether MHMO influenced the survival of normal cells using a methyl thiazolyl tetrazolium (MTT) assay. Even at concentrations as high as 600 µg mL^−1^, the 3T3 cell viability remained above 90%, suggesting that MHMO had minimal cytotoxicity toward normal cells (Figure S12a, Supporting Information). The same experiment was performed to estimate the antitumor effect of MHM and MHMO on CT26 cells (MSS‐type mouse colon cancer cells) with or without US irradiation (Figure S12b, Supporting Information). These results revealed that the US alone did not cause significant cell death. A statistically significant and dose‐dependent upregulation of cell death was detected upon exposure to increasing concentrations of MHM and MHMO.

Remarkably, a concentration of 600 µg mL^−1^ MHMO combined with US led to a cell inhibition rate exceeding 70%. We co‐cultured FITC‐labelled MHMO with CT26 cells at 37 °C for varying durations (0, 0.5, 2, and 4 h). Subsequently, flow cytometry and Bio–TEM were used to assess the uptake of MHMO by CT26 cells (Figure 3b; Figure S13, Supporting Information), which revealed that MHMO uptake by the cells began after 0.5 h of co‐culturing. Using ICP for analytical quantification, we measured the increase in intracellular Ca^2+^ and Mg^2+^ concentrations across the different treatment groups. Our findings revealed that in the MHM and MHMO groups, the mean fold increases in Ca^2^⁺ concentrations were 7.3 and 8.1, respectively, while the corresponding increases for Mg^2^⁺ were 7.6 and 7.9. Notably, the application of ultrasonic‐assisted treatment markedly enhanced these proliferation rates, elevating the average fold increases in Ca^2^⁺ concentrations in the MHM and MHMO groups to 15.2 and 18.1, respectively, and those corresponding to Mg^2^⁺ to 16.6 and 17.3.

A high concentration of calcium in the cytosol enters the mitochondria via voltage‐dependent anion channels (VDAC) or calcium uniporters, stimulating respiratory chain activity and resulting in an elevated amount of ROS.^[^ ^21^ ^]^ The non‐fluorescent compound 2ʹ,7ʹ‐dichlorodihydrofluorescein diacetate (DCFH‐DA) can be oxidized by ROS to form dichlorofluorescein (DCF), which emits solid green fluorescence.^[^ ^22^ ^]^ Therefore, DCFH‐DA served as a probe to detect intracellular ROS (Figure 3c). The results demonstrated that neither the PBS nor the stand‐alone US group generated fluorescence, whereas the green fluorescence intensity gradually increased from the MHM to the MHMO + US group, indicating an increasing trend in ROS production, particularly under piezoelectric catalysis. We propose a significant enhancement in the crosstalk between Ca^2+^ and ROS due to piezoelectric effects. Consequently, we used the Ca^2+^ fluorescent probe Fluo‐4 Acetoxymethyl Ester (Fluo‐4 AM) to assess intracellular Ca^2+^ levels (Figure 3d; Figure S14, Supporting Information). The results demonstrated that the MHM and MHMO groups exhibited increased fluorescence intensity compared to the PBS and US groups, with the MHMO + US group exhibiting the strongest fluorescence intensity. This indicates that MHMO is capable of releasing some Ca^2+^ in the acidic tumor microenvironment, with the piezoelectric effect further amplifying this release, leading to calcium overload.

### 2.4 MHMO Induces Necroptosis Leading to Colon Cancer Cell Death

Perturbations in cellular Ca^2+^ homeostasis and increased ROS levels can lead to the upregulation of the death receptor DR5, subsequently activating DR5‐mediated cell death pathways such as apoptosis and necroptosis.^[^ ^6^ ^]^ However, apoptosis serves as the primary defense mechanism against tumor progression and is often suppressed in several tumors.^[^ ^23^ ^]^ Under these conditions, necroptosis emerges as an alternative protective mechanism, exerting significant tumoricidal effects. Immunofluorescence revealed the post‐treatment cell survival conditions in different groups, as assessed using calmodulin (AM) and propidium iodide (PI) (Figure S15, Supporting Information). Minor cellular damage was observed in the MHM and MHMO groups, whereas the MHMO + US group exhibited the most substantial cell injury. This result revealed that calcium overload induced by MHMO led to tumor cell damage, whereas a substantial increase in ROS production, influenced by the piezoelectric effect, enhanced cell death. Subsequently, the mitochondrial membrane potential indicator J‐aggregate‐forming lipophilic cation 1 (JC‐1) was used to characterize the cell viability (Figure 3e; Figure S16, Supporting Information). Flow cytometry data showed an increase in monomeric green fluorescence within the MHM and MHMO groups, whereas, under the influence of piezoelectric catalysis and calcium overload, the MHM + US and MHMO + US groups exhibited a more pronounced decrease in membrane potential, leading to a significant increase in cellular apoptosis. Flow cytometry was also conducted to determine the cellular apoptosis status among the different treatment groups (Figure S17, Supporting Information). The apoptosis rate increased from 16.74% to 54.73%. Therefore, MHMO + US is more likely to induce tumor cell death by triggering necroptosis. Subsequently, we employed and quantitatively analyzed western blotting to investigate the occurrence of necroptosis as a backup mechanism for apoptosis (Figure 3f; Figure S18, Supporting Information). The results showed a slight increase in the phosphorylation of RIPK1, RIPK3, and MLKL in both MHM and MHMO groups. Upon piezoelectric catalysis, this increase became more significant, whereas the total expression levels of RIPK1, RIPK3, and MLKL remained unchanged. These observations suggest the occurrence of necroptosis. The expression of the apoptotic markers Bax and Bcl2 was consistent with our previous observations of apoptosis. Crucially, we detected a mild elevation in DR5 expression in both the MHM and MHMO groups, whereas a more substantial upregulation was observed in the MHM + US and MHMO + US groups. This suggests that piezoelectric catalysis promoted by HPA effectively released Ca^2+^ and ROS, consequently promoting DR5 upregulation and facilitating the activation of cell death pathways. We administered the apoptosis inhibitor Z‐VAD‐fmk and necroptosis inhibitor necrostatin‐1 (Nec‐1) separately to CT26 cells treated with MHMO + US (Figure S12c, Supporting Information). The results indicated that Z‐VAD‐fmk partially suppressed apoptosis and facilitated the transition to necroptosis, whereas Nec‐1 concurrently blocked both death modalities. This further confirmed that necroptosis could serve as a backup cell death mechanism to overcome the apoptosis resistance induced by MHMO + US. To directly observe the mode of cell death following drug treatment, we employed biological TEM (bio‐TEM) to assess CT26 cells co‐cultured with MHMO (Figure 3g). The results revealed that following drug administration, only a few cells underwent apoptosis (evidenced by cell shrinkage and apoptotic body formation).^[^ ^24^ ^]^ while more cells displayed necroptotic features (characterized by organelle swelling, plasma membrane rupture, and cytoplasmic vacuolation).^[^ ^25^ ^]^

### 2.5 Evaluation of DC Maturation Induced by ICD Stimulation

Necroptosis is a highly proinflammatory mode of cell death that is more immunogenic than apoptosis.^[^ ^26^ ^]^ In addition to the specific cytotoxic effects of necroptosis, ICD can induce the release of damage‐associated molecular patterns (DAMPs) following cell death. This subsequently attracts and activates dendritic cells (DCs) and ultimately leads to the reprogramming of the tumor immune microenvironment.^[^ ^27^ ^]^ During the ICD process, the principal components released as DAMPs include the “eat me” signal calreticulin (CRT) and high‐mobility group box‐1 (HMGB1).^[^ ^28^ ^]^ We employed immunofluorescence to evaluate the expression of CRT and HMGB1 in the various treatment groups (Figure 3h,i). A slight increase in CRT expression and a minor reduction in nuclear HMGB1 levels were observed in the MHM and MHMO groups compared with the PBS, US, and single‐agent groups, with this trend becoming more pronounced with piezoelectric catalysis. Simultaneously, the western blot assessment of HMGB1 expression was consistent with the immunofluorescence findings (Figure 3j; Figure S19, Supporting Information). These findings demonstrated that MHMO‐induced necroptosis leads to ICD, particularly in the presence of piezoelectric catalysis.

**Figure 4:**
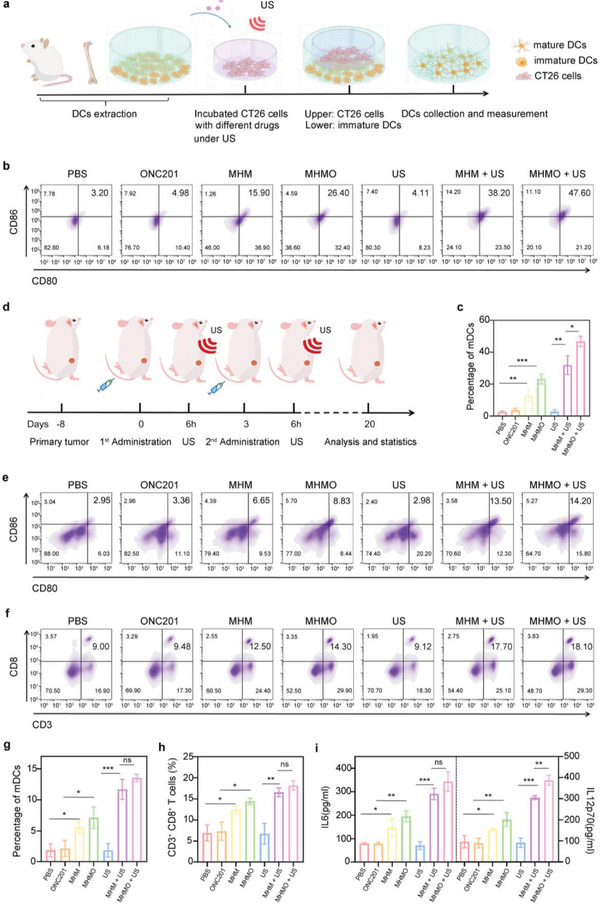
MHMO induces TME reprogramming and stimulates immune responses both in vitro and in vivo. a) The diagram illustrating the process of isolating mononuclear cells from mouse femurs, inducing them into immature dendritic cells (DCs), followed by co‐culturing with post‐treatment CT26 cells. b) Mature DCs (expressing CD86^+^ and CD80^+^) were identified using flow cytometric analysis. c) Statistical analysis of DCs maturation across various treatment groups in vitro. d) The diagram illustrates the process of administering treatment following the unilateral inoculation of a tumor in mice. e) Staining and flow cytometric analysis of isolated splenic immune cells in mice for evaluating DCs maturation across different treatment groups. f) The proportion of CD8^+^ T cells in spleens from various treatment groups was assessed using flow cytometry. g) Statistical analysis of DCs maturation across various treatment groups in vivo. h) Assessment of CD8^+^ T cells in vivo. i) ELISA was used to measure cytokine (IL‐6 and IL‐12p70) secretion levels under various treatments. n.s. indicates no significance, ^*^ *p* < 0.05, ^**^ *p* < 0.01, ^***^ *p* < 0.001, and ^****^ *p* < 0.0001.

DAMPs mediate the recruitment and engulfment of phagocytic cells, which ingest dying cells and subsequently present their antigens to the surfaces of DCs.^[^ ^4^, ^29^ ^]^ Activated DCs migrate to immune organs and differentiate into mature DCs.^[^ ^30^ ^]^ To confirm the effectiveness of MHMO + US‐induced necroptosis in enhancing immune responses within tumor cells, we co‐cultured bone marrow‐derived dendritic cells (BMDCs) with pretreated CT26 cells (**Figure** **4**a). Flow cytometry was used to evaluate DC maturation using the CD11c^+^, CD80^+^, and CD86^+^ markers (Figure 4b,c). DC maturation was elevated in the MHM‐ and MHMO‐treated CT26 cells compared with the PBS, US, and single‐agent groups, with a more pronounced enhancement of DC maturation observed following stimulation with MHMO + US. This suggests that piezoelectric‐catalyzed MHMO can induce necroptosis and effectively stimulate ICD, subsequently inducing DC maturation.

Subsequently, we explored the induction of DC maturation in the different groups in vivo. To investigate the pharmacokinetic properties of MHMO, we intravenously administered MHMO through the tail vein of mice and evaluated the distribution of Ca^2+^ and Mg^2+^ in the major organs at various time points (Figure S20, Supporting Information). Our results indicated that the optimal therapeutic time point for MHMO administration was 6 h, with MHMO preferentially enriched in the liver and spleen, which are recognized for their richness in the reticuloendothelial system. Using the fluorescent signal from Cyanine5.5 (Cy5.5), accurate monitoring of the targeted delivery of MHMO could be achieved through fluorescence imaging guidance in mice (Figure S21, Supporting Information). The results in Figure S22 (Supporting Information) demonstrate that the blood circulation of MHMO NPs adheres to the classic two‐compartment model, with the respective half‐lives calculated as τ_1/2α_ = 0.29 h and τ_1/2β_ = 5.89 h. This characteristic enhances the effective clearance of MHMO NPs from normal organs and facilitates their aggregation in tumors. The results demonstrated that following intravenous injection, the fluorescent signal peaked at 6 h and subsequently reached its lowest point at 48 h. Consequently, the optimal treatment time was 6 h post‐intravenous injection. In light of these observations, we inoculated CT26 cells into the right dorsal side of mice. After one week, the mice were haphazardly divided into distinct groups and treated via tail vein injection. US therapy was performed 6 h after the administration (Figure 4d). Mice were euthanized 20 days post‐administration, and their spleens were harvested for flow cytometry analysis. Both the MHM and MHMO groups exhibited increased DC maturation rates, with the highest rate observed in the MHMO + US group, consistent with the observations in vitro (Figure 4e,g). Additionally, the MHMO + US group displayed significantly elevated levels of IL‐12p70 (an antigen presentation‐related cytokine)^[^ ^31^ ^]^ and IL‐6 (a proinflammatory cytokine).^[^ ^32^ ^]^ (Figure 4i). These results suggest that MHMO‐induced necroptosis under Ca^2+^, Mg^2+^, and piezoelectric stimulation in an acidic tumor environment can promote DC maturation, consequently leading to the generation of a proinflammatory tumor microenvironment.

### 2.6 MHMO‐Induced TME Remodeling to Activate Antitumor Immune Responses In Vivo

Following ICD in tumor cells, DAMPs stimulate DC maturation and macrophage polarization, subsequently leading to T cell activation and initiating an immune response.^[^ ^28a^ ^]^ Additionally, the costimulatory molecule LFA‐1 in CD8^+^ T cells requires Mg^2+^ to regulate its bent conformation, thereby increasing calcium flux, facilitating signal transduction, and promoting cytotoxicity.^[^ ^8^ ^]^ Based on our observations, we postulate that MHMO not only promotes immune responses by inducing necroptosis in tumor cells but also enhances T‐cell activation through Mg^2+^ release. Therefore, spleens were isolated and harvested from the previously mentioned murine model, and the CD8^+^ T cell population was quantified by flow cytometry (Figure 4f,h). Compared to the PBS, US, and single‐agent treatment groups, the proportion of CD8^+^ T cells was significantly elevated in the MHM and MHMO groups, reaching its highest level after US administration.

**Figure 5:**
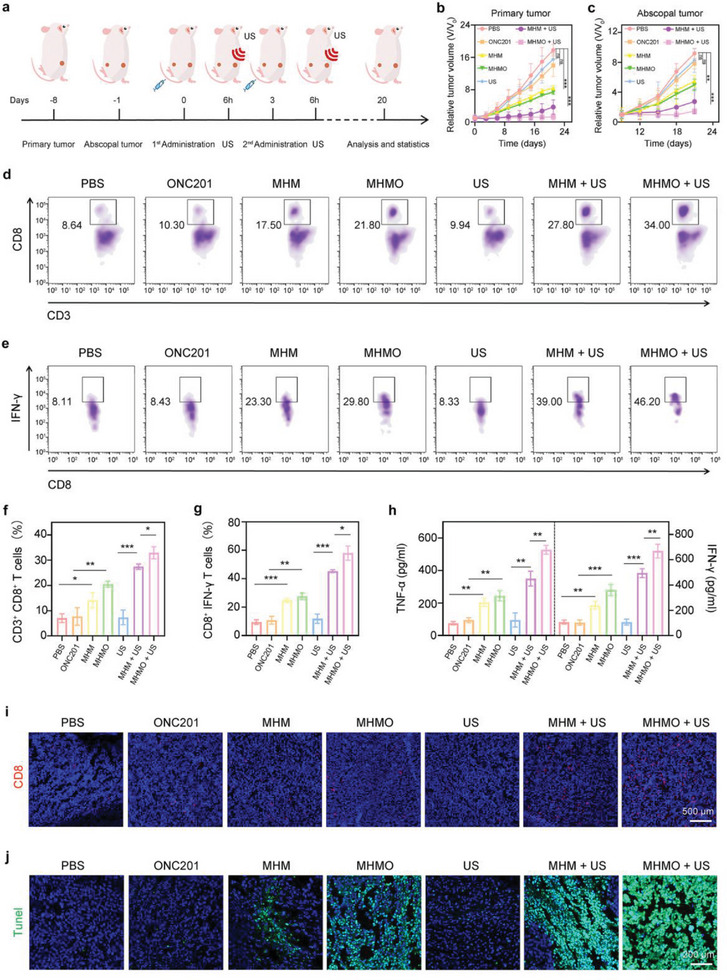
Assessment of the effectiveness of xenograft treatment and activation of the immune system in vivo. a) Experimental design for the management of bilateral tumors. b, c) BALB/c mice received implants of CT26 cells (*n* = 5). Tumor dimensions were recorded at three‐day intervals. The tumor mass was evaluated 20 days post‐establishment of the tumor model and commencement of treatment. b) Progression of the initial tumor and c) progression of the abscopal tumor. d) Isolation and preparation of a cell suspension from the primary tumor site in post‐treatment mice. Subsequently, staining of these cells was performed to identify CD8^+^ T cells (gating on CD45^+^) for analysis via flow cytometry. e) The proportion of cytotoxic T lymphocytes (CTLs) expressing IFN‐γ^+^ CD8^+^ in tumor tissues from different treatment groups. f) In vivo quantification of CD8^+^ T cells. g) In vivo quantification of CTLs. h) ELISA was used to quantify the expression levels of tumor necrosis factor‐alpha (TNF‐α) and IFN‐γ cytokines in mouse serum. i) Fluorescence microscopy revealed the presence of CD8^+^ T lymphocyte staining within the primary tumor tissue. The scale bar corresponds to a length of 500 µm. j) Primary tumor samples underwent TUNEL staining analysis. The scale bar represents 200 µm. n.s. indicates no significance, ^*^ *p* < 0.05, ^**^ *p* < 0.01, ^***^ *p* < 0.001, and ^****^ *p* < 0.0001.

To more accurately determine the impact of MHMO on the growth conditions of primary tumors and distant metastases, we inoculated CT26 cells (5 × 10^5^ cells/mouse) into the right dorsal side of mice and performed contralateral inoculation (2.5 × 10^5^ cells/mouse) one week later. After 24 h, the mice were randomly assigned to seven groups, designated as the PBS, ONC201, MHM, MHMO, US, MHM + US, and MHMO + US groups. Following intravenous administration, primary tumors were subjected to US irradiation at 6 h post‐injection. After euthanization on day 20, primary and metastatic tumor specimens were collected for subsequent experiments (**Figure** **5**a). Gross and ex vivo anatomical images of mouse tumors revealed the tumor sizes in different groups following treatment (Figure S23, Supporting Information). The bilateral tumor growth curves revealed that the tumor volumes in the MHM and MHMO groups were slightly reduced compared with those in the control group, and this volume reduction became more pronounced following piezoelectric catalysis (Figure 5b,c). These outcomes were more pronounced in the metastatic group, suggesting a potential association between Mg^2+^ release and the inhibition of distant colorectal cancer metastasis.^[^ ^33^ ^]^ Subsequently, we quantified the number of infiltrating immune cells at primary and metastatic sites. Notably, effector T (CD45^+^, CD3^+^, and CD8^+^) and cytotoxic T cells (CD3^+^, CD8^+^, and IFN‐γ^+^) were markedly elevated in the MHM and MHMO groups at the primary location, with infiltration further accentuated following piezoelectric stimulation (Figure 5d–g). Similar findings were observed for distant metastatic tumors (Figure S24, Supporting Information).

Serum samples were also collected from the mice to assess cytokine secretion (Figure 5h). Enzyme‐linked immunosorbent assay (ELISA) analysis revealed a marked elevation in tumor necrosis factor‐alpha (TNF‐α) and IFN‐γ concentrations post‐treatment, which was consistent with the findings from flow cytometry. These data imply an enhanced secretion of proinflammatory cytokines by immune cells following drug administration, which further increased substantially with piezoelectric stimulation. Immunofluorescence confirmed the infiltration of CD8 cells into tumor tissues across various therapeutic groups (Figure 5i). Terminal deoxynucleotidyl transferase dUTP nick‐end labeling (TUNEL) and hematoxylin and eosin (H&E) staining were also used to evaluate the treatment outcomes in various groups, demonstrating a positive correlation between tumor cell death and CD8^+^ T cell infiltration (Figure 5j; Figure S25, Supporting Information). These observations indicate that piezoelectric stimulation enhances MHMO‐driven necroptotic cell death and Mg^2+^ release, contributing to the remodeling of the tumor microenvironment and immune responsiveness.

**Figure 6:**
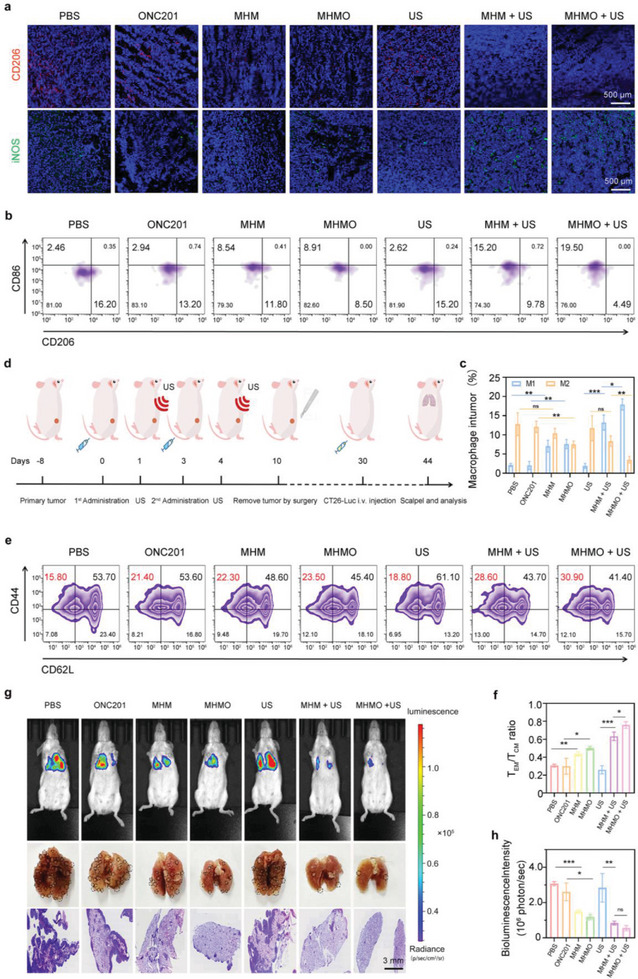
The establishment of long‐term immunological memory and augmentation of immune cell activation through MHMO‐mediated mechanisms. a) Immunofluorescence evaluation of M2‐ (CD206^+^) and M1‐type (iNOS^+^) macrophage infiltration in primary tumor tissue sections. The scale bar represents 500 µm. b) Flow cytometric evaluation was conducted to determine the proportions of M1‐like (CD86^+^) macrophage and M2‐like (CD206^+^) macrophage (gating F4/80^+^ and CD11b^+^) infiltration in the primary tumor. c) Quantitative evaluation of the M1/M2 ratio in the microenvironment of the primary tumor. d) Schematic representation of the efficacy of MHMO + US treatment on tumor lung metastasis. e) Flow cytometry data results and f) quantitative statistical analysis of Tem (CD8^+^, CD44^+^, and CD62L^–^)/Tcm (CD8^+^, CD44^+^, and CD62L^+^) cells in the spleen. g) Lung metastasis imaging of mice from different treatment groups on day 14, whole lung images (with circles representing lung nodules), and H&E staining of lung tissue. n.s. indicates no significance, ^*^ *p* < 0.05, ^**^ *p* < 0.01, ^***^ *p* < 0.001, and ^****^ *p* < 0.0001.

The role of macrophages as another pivotal element within the immune microenvironment has attracted our attention.^[^ ^34^ ^]^ M1‐polarized macrophages exert proinflammatory effects, thereby directly inducing tumor cell death and indirectly enhancing T cell‐mediated antitumor responses. M2‐polarized macrophages typically exhibit anti‐inflammatory functions and are generally the predominant type of tumor‐associated macrophages (TAMs) during tumor progression.^[^ ^35^ ^]^ Their involvement in establishing an immunosuppressive microenvironment leads to the development of resistance to immunotherapeutic approaches.^[^ ^36^ ^]^ Previous studies have confirmed that piezoelectric signals can induce macrophage reprogramming by influencing M1 macrophage polarization.^[^ ^37^ ^]^ Owing to the remarkable piezoelectric properties of MHMO demonstrated in previous experiments, we investigated its potential influence on macrophage polarization in the tumor microenvironment. Immunofluorescence analysis was performed to identify the M1 polarization‐associated marker iNOS (responsible for eliciting NO production and subsequent inflammatory responses)^[^ ^38^ ^]^ and the M2 polarization‐associated marker CD206 in mouse tumor tissues (**Figure** **6**a). Our findings indicate that macrophages in the PBS, ONC201, and US groups primarily exhibited CD206 expression, whereas iNOS expression remained notably low. Enhanced iNOS^+^ M1‐like macrophage and reduced CD206^+^ M2‐like macrophage levels were observed in the MHM and MHMO groups, potentially linked to M1 polarization induced by ROS release. The application of piezoelectric stimulation in the MHM and MHMO groups resulted in a marked elevation in M1‐polarized macrophages and a substantial decrease in M2‐polarized macrophages in the tumor microenvironment. Flow cytometric evaluation of M1 (F4/80^+^, CD11b^+^, and CD86^+^) and M2 (F4/80^+^, CD11b^+^, and CD206^+^) macrophage proportions in primary tumor tissues substantiated a transition from M2 to M1 polarization in the MHM and MHMO groups subjected to piezoelectric stimulation, which was consistent with our immunofluorescence findings (Figure 6b,c). Correspondingly, the proportion of M1 to M2 cells in distant metastatic tumors was evaluated via flow cytometry, which yielded results corresponding to those observed in the initial tumor tissues (Figure S26, Supporting Information).

### 2.7 T Cell Memory Induction and Lung Metastasis Inhibition

Mg^2+^ release enhances memory CD8^+^ T cell‐mediated antitumor immunity.^[^ ^8^ ^]^ To investigate long‐term T‐cell memory responses in mice, we treated mice inoculated with CT26 tumor cells and subsequently performed surgical excision. A lung metastasis model was established by inoculating mice with luciferase‐expressing CT26 (CT26‐luc) tumor cells via tail vein injection after one month (Figure 6d). 14 days post tail vein injection, spleen‐derived lymphocytes were collected for flow cytometric examination (see Figure 6e,f). In the MHM and MHMO groups, there was a notable rise in the relative ratios of effector memory T cells (characterized as CD3^+^, CD8^+^, CD44^+^, CD62L^–^, Tem) and central memory T cells (identified as CD3^+^, CD8^+^, CD44^+^, CD62L^+^, Tcm), with these increases becoming more marked following piezoelectric stimulation. Bioluminescence and H&E staining were used to directly assess lung metastasis in mice 14 days after model establishment (Figure 6g,h). The results revealed that the untreated control mouse group exhibited a significant increase in lung fluorescence intensity, accompanied by respiratory distress and hemoptysis symptoms. Moreover, a notable increase in ex vivo metastatic lung nodules was observed. Mice subjected to MHMO + US treatment showed no significant differences in body weight changes (Figure S27, Supporting Information) and exhibited favorable health, with both bioluminescent and ex vivo evaluations revealing a significantly reduced number of pulmonary metastatic nodules compared to the control group.

These findings indicate that MHMO + US treatment can generate long‐term immune memory, effectively suppressing tumor metastasis and recurrence. In addition, no substantial damage was observed following H&E staining of additional organs (the heart, liver, spleen, and kidneys) in the various treatment groups (Figure S28, Supporting Information). To ensure the biosafety of MHMO, we collected blood samples from healthy mice at distinct post‐intravenous injection time intervals to evaluate the hematological parameters and blood biochemistry; no notable abnormalities were detected in the hematological analysis compared to the control group (Figure S29, Supporting Information).

### 2.8 Identification of Therapeutic Molecular Mechanisms through RNA‐Seq Analysis

**Figure 7:**
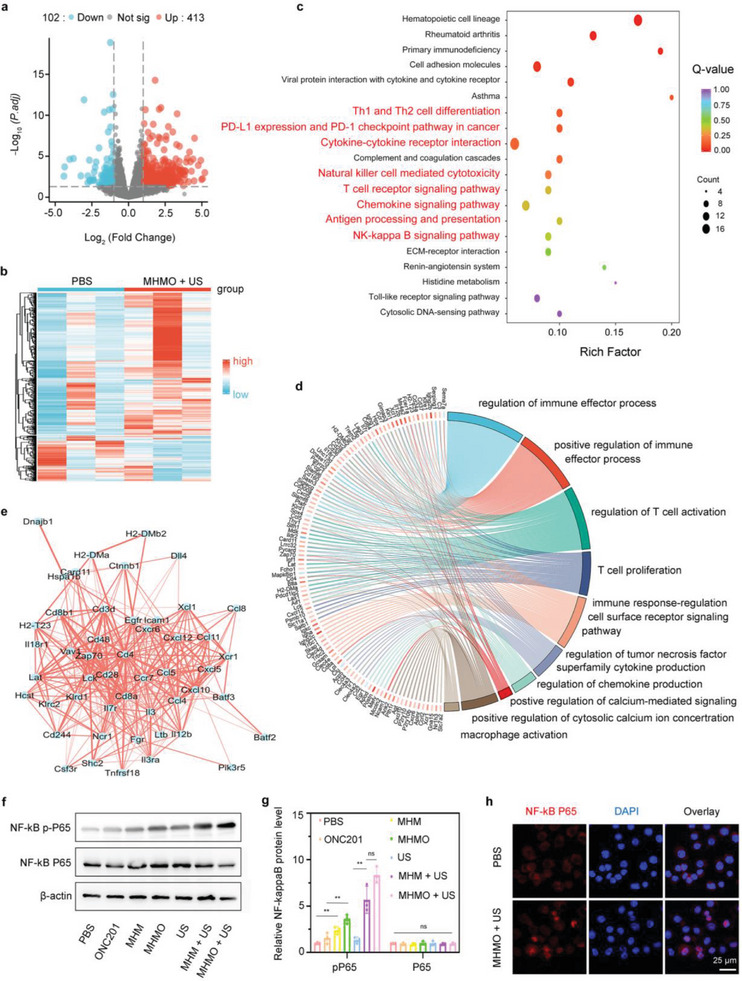
RNA sequencing investigation revealing the underlying processes of MHMO + US therapy. a) A graphical representation through a volcano plot and b) a heatmap display illustrates the alterations in mRNA expression profiles of genes with differential expression post‐treatment. c) Kyoto Encyclopedia of Genes and Genomes (KEGG) analysis reveals the 20 most significantly enriched pathways in response to MHMO + US therapy. d) Gene ontology (GO) circular visualization elucidating the biological processes and functional implications of differentially expressed genes subsequent to MHMO + US therapy. e) Interactome analysis of variably expressed immune‐associated proteins. f) Western blot analysis demonstrating the activation status of the NF‐κB pathway in different treatment groups. g) Quantitative statistical analysis of p‐P65 protein expression. h) Immunofluorescence showing P65 nuclear translocation following MHMO + US treatment. The scale bar represents 25 µm.

To further investigate the antitumor and immune‐activating mechanisms of MHMO in colon cancer, we extracted tumor tissues from mice treated with MHMO + US and PBS for RNA‐Seq analysis. Volcano plot and heatmap analyses revealed that following MHMO + US treatment, the mRNA expression of 413 genes, including those for CD28, CD37, CD8a, Ccl5, and IL12b, was upregulated, while the expression of 102 genes, including those for Fn1, Egr1, Sema7a, and Sox4, was downregulated (**Figure** **7**a,b). The TCR costimulatory receptor, CD28, is a key target of PD‐1 blockade, and T cells with high CD28 expression are likely to enhance the efficacy of immunotherapy.^[^ ^39^ ^]^ Recently, CD37 has emerged as an emerging target that influences antitumor immune responses by regulating B and T cell activation, proliferation, and survival. Concurrently, as a death receptor, CD37 induces neoplastic cell death following antibody binding.^[^ ^40^ ^]^ CD8, especially tumor‐infiltrating CD8, is an important indicator of tumor prognosis, therapeutic efficacy, and immune response.^[^ ^41^ ^]^ Similarly, the upregulation of cytokines and chemokines, including Ccl5 and IL12b,^[^ ^42^ ^]^ indicates an increase in antigen presentation and immune responses following treatment, which is consistent with our previous ELISA results. As a component of the extracellular matrix, a reduction in Fn1 expression can contribute to the inhibition of tumor cell metastasis. Moreover, studies have indicated a direct relationship between Fn1 expression levels and the number of M2 macrophages.^[^ ^43^ ^]^ This closely aligns with our previous observation that MHMO promotes macrophage polarization from M2 to M1 under piezoelectric stimulation. Furthermore, the tumor cell stemness and drug resistance markers Egr1, Sema7a, and Sox4 exhibited reduced expression, suggesting that MHMO + US treatment inhibited tumor cell metastasis.^[^ ^44^ ^]^ This is associated with Mg^2+^ release and the generation of long‐term memory T cells.

Subsequently, we performed Kyoto Encyclopedia of Genes and Genomes (KEGG) and Gene Ontology (GO) enrichment analyses to explore the pathways and biological processes associated with the differentially expressed genes (Figure 7c,d). The KEGG results revealed that after MHMO + US treatment, several immune‐positively correlated pathways, such as Th1 and Th2 cell differentiation, PD‐L1 expression, the PD‐1 checkpoint pathway in cancer, cytokine‐cytokine receptor interaction, and natural killer cell‐mediated cytotoxicity, were significantly enriched. Activation of the “T‐cell receptor signaling pathway” suggests that Mg^2+^ release alters the conformation of CD8^+^ T‐cell receptors, promoting the formation of immune synapses and signal transduction. Additionally, the activation of the “Antigen processing and presentation” pathway confirms that MHMO + US treatment induces necroptosis in tumor cells and promotes the occurrence of ICD and the maturation of DCs. Notably, our observations reveal that the NF‐κB signaling pathway was activated following MHMO + US treatment. The NF‐κB signaling pathway represents a classic inflammation‐associated pathway, instrumental in promoting the M1 polarization of TAMs. Consequently, we propose that MHMO + US treatment promotes M1 polarization through the mediation of the NF‐κB signaling pathway, ultimately increasing T‐cell activation. GO biological function enrichment analysis also demonstrated the positive regulation of immune effector processes such as T cell activation, proliferation, macrophage activation, regulation of cell surface receptor signaling pathways, and regulation of chemokine production. Enrichment of the biological functions “positive regulation of cytosolic calcium ion concentration” and “regulation of tumor necrosis factor superfamily cytokine production” also suggests that MHMO + US treatment leads to the intracellular release of Ca^2+^ and the production of tumor necrosis factors following the therapy. Based on the protein‐protein interaction network of these differentially expressed genes related to the immune response, we identified key hub proteins, including CD28, CD8a, and Ccl5 (Figure 7e), which are essential for mediating the corresponding immune responses.

Subsequently, we validated that MHMO + US induced M1 polarization through the NF‐κB signaling pathway. Following the co‐culture of RAW264.7 mouse macrophages with CT26 cells from the various treatment groups for 48 h, we performed a western blotting analysis (Figure 7f,g). Our findings revealed a marked elevation in P65 protein phosphorylation in the MHMO + US group, whereas the overall protein levels remained unaltered. Similarly, the immunofluorescence results demonstrated an increase in P65 nuclear translocation in the MHMO + US‐treated group compared to that in the PBS control group (Figure 7h; Figure S30, Supporting Information). This suggests that MHMO induces the upregulation of death receptor DR5 through the release of Ca^2+^, thereby promoting necroptosis and ICD in tumor cells. Meanwhile, piezoelectric stimulation can lead to the activation of the NF‐κB signaling pathway in macrophages, resulting in M1 polarization. The release of Mg^2+^ induces active conformations in T cell receptors, enabling them to recognize and respond to signals from DCs and macrophages, ultimately promoting the formation of cytotoxic T lymphocytes (CTLs) and memory T cells.

## 3 Conclusion

In summary, we synthesized Mg^2+^‐doped HAP using a hydrothermal method. Mg/HAP was surface‐coated with MSN to facilitate the loading of ONC201, an agonist ligand targeting the DR5 death receptor, onto tumor cells. The final synthesized therapeutic system was named MHMO. Our investigation reveals for the first time that MHMO can facilitate substantial ROS release under piezoelectric catalysis. Simultaneously, Ca^2+^ release acts synergistically with ROS to stimulate necroptosis in tumor cells, thereby overcoming apoptosis resistance. Tumor cells undergoing necroptosis can promote ICD and induce DC maturation both in vitro and in vivo. Simultaneously, in the tumor microenvironment, MHMO + US promotes M1‐type macrophage polarization. Concurrently, the released Mg^2+^ enhances T‐cell receptor activation and facilitates the immune response. Ultimately, this piezoelectrically catalyzed dual‐targeting system has the potential to suppress the recurrence and metastasis of tumor cells, providing a new therapeutic strategy for immunotherapy.

## 4 Experimental Section

### Animal Experiments

Female 4‐5‐week‐old BALB/c mice were purchased from Beijing Vital River Laboratory Animal Technology Co., Ltd. All the mice were maintained in animal facilities under pathogen‐free conditions, and the animal study was approved by the Animal Ethical and Welfare Committee (AEWC) of the Second Affiliated Hospital of Harbin Medical University (Harbin, China). The authors strictly adhered to the ethical guidelines for animal care and use as outlined in the Guidelines of the Drug Safety Evaluation Center of Harbin Medical University (No. SYDW 2019–82).

## Conflict of Interest

The authors declare no conflict of interest.

## Author Contributions

J.Y. and Y.D. contributed equally to this work. The manuscript was written with contributions from all authors. All the authors approved the final version of the manuscript.

## Supporting information

Supporting Information

## Data Availability

Research data are not shared.
