# Supplementary material for: Employing Piezoelectric Mg2+‐Doped Hydroxyapatite to Target Death Receptor‐Mediated Necroptosis: A Strategy for Amplifying Immune Activation
Source: Adv Sci (Weinh). 2024 Jan 22;11(13):2307130. doi: 10.1002/advs.202307130 (PMC10987113; doi:10.1002/advs.202307130)
Supplement: Supplementary file 1 — Supporting Information [file ADVS-11-2307130-s001.pdf]

ADVANCED SCIENCE
Open Access

Supporting Information

for *Adv. Sci.*, DOI 10.1002/advs.202307130

Employing Piezoelectric Mg^{2+}-Doped Hydroxyapatite to Target Death Receptor-Mediated Necroptosis: A Strategy for Amplifying Immune Activation

Jiani Yang, Yaqian Du, Yuanfei Yao, Yuanyu Liao, Bojun Wang, Xuefan Yu, Kaikun Yuan, Yanqiao Zhang, Fei He* and Piaoping Yang**

Supporting Information

Employing Piezoelectric Mg^{2+}-Doped Hydroxyapatite to Target Death Receptor-Mediated Necroptosis: A Strategy for Amplifying Immune Activation

Jiani Yang, Yaqian Du, Yuanfei Yao, Yuanyu Liao, Bojun Wang, Xuefan Yu, Kaikun Yuan, Yanqiao Zhang, Fei He*, and Piaoping Yang**

Experimental Section

Chemicals and Reagents. All the chemicals were used without additional purification steps. Anhydrous calcium chloride ($CaCl_2$), magnesium chloride ($MgCl_2$), and anhydrous sodium dihydrogen phosphate (NaH_2PO_4) were obtained from the Tianjin Guangfu Fine Chemical Research Institute (Tianjin, China). Ethylenediamine, cetyltrimethylammonium bromide (CTAB), cyclohexane, isopropanol, and tetraethyl orthosilicate (TEOS) were obtained from Aladdin (Shanghai, China). 3,3′,5,5′-tetramethyl-benzidine (TMB), 1,3-diphenylisobenzofuran (DPBF), 2,2′,6,6′-tetramethylpiperidine (TEMP), and 2,7-dichlorofluorescein diacetate (DCFH-DA) were obtained from Aladdin (Shanghai, China). MTT, 5,5-dimethyl-1pyrroline N-oxide (DMPO), 4′,6-diamidino-2-phenylindole (DAPI), calcein-AM, propidium iodide (PI) were procured from Beyotime (Shanghai, China), and Annexin V-FITC Apoptosis Kit from Solarbio (Beijing, China). D-Luciferin potassium was from Meilunstar (Dalian, China) Mouse IL-6, TNF-α, IFN-γ, and IL-12p70 ELISA kits were obtained from Shanghai Enzyme Link Biotechnology Co., Ltd. (Shanghai, China). An H&E staining kit was procured from Beijing Solarbio Science & Technology Co., Ltd. (Beijing, China).

Characterization. The morphology of the TEM images of the synthesized MHMO NPs was analyzed using an FEI Tecnai G^2 S-Twin transmission electron microscope. X-ray diffraction (XRD) images of the MHMO NPs were obtained using a Rigaku D/max-TTR-III. The chemical compositions of Ca, Mg, P, Si, and O in the MHMO NPs were determined by an ESCALAB 250Xi X-ray photoelectron spectroscopy (XPS). The spectral absorbances of the samples in the UV-visible range were quantified using a UV-vis spectrophotometer (Shimadzu UV-1601). A Bruker EMX1598 spectrometer was employed to acquire electron-spin-resonance (ESR) spectra to identify $\cdot O_2^-$, $^1O_2$, and $\cdot OH$. A Leica TCS SP8 confocal microscope was used to

capture Confocal Laser Scanning Microscopy (CLSM) images. Protein extraction from the cells was followed by separation using 8%–12% SDS-PAGE and transfer onto a polyvinylidene fluoride (PVDF) membrane (Merck, Darmstadt, Germany). Flow cytometric analyses were performed using a BD FACSMeldoy flow cytometer.

Synthesis of Mg/HAP. First, HAP was produced using a hydrothermal method. Anhydrous $CaCl_2$ (3.8 mmol), $MgCl_2$ (0.2 mmol), and anhydrous NaH_2PO_4 (2.4 mmol) were separately dissolved in 20 mL of deionized water. Subsequently, the $CaCl_2$ and $MgCl_2$ solutions were titrated into the NaH_2PO_4 solution, and the mixture was stirred for 0.5 h to achieve uniformity. Following this, the pH value of the combined solution was adjusted to a value of 12 using ethylenediamine. The resultant suspension was decanted into a Teflon-sealed autoclave and subjected to a thermal protocol at 200 ℃ for 24 h. Upon cooling to ambient temperature, the obtained sediment was harvested and washed with deionized water and ethanol multiple times for purification. Finally, a drying phase at a constant 60 °C over a 24-h duration yielded the pristine HAP/Mg.

Synthesis of MHM. Under ultrasonication, Mg/HAP, 1.2 g of urea, and 2 g of cetyltrimethylammonium bromide (CTAB) were incorporated into 60 mL of deionized water. Once a clear and transparent mixed solution was obtained, it was transferred to a three-necked flask. Subsequently, cyclohexane (60 mL), isopropanol (2 mL), and tetraethyl orthosilicate (TEOS) (1 mL) were added to the solution. The combined solution was then subjected to mechanical stirring to ensure homogeneity and maintained at 75 °C for a period of 16 h. During the initial 2 h, the three-necked flask air stopper was unsealed to allow cyclohexane evaporation and was subsequently sealed again. The nanoparticles were harvested via centrifugation and purified by washing with deionized water and ethanol. The residual CTAB was eliminated using an NH_4NO_3-infused ethanol solution, sustained at 65 °C for a 24-h duration. The final product was obtained via centrifugation, purged with water and ethanol, and subsequently dried in an oven at 60 °C.

Synthesis of MHMO. MHM (10 mg) and ONC201 (5 mg) were combined in 5 mL of methanol and magnetically stirred for a 24-h duration. Subsequently, the final product was isolated by centrifugation and subjected to three methanol wash cycles. The supernatant of the product was collected to evaluate the unloaded ONC201 by measuring the absorbance at a wavelength of 388 nm. The loading capacity (LC) and encapsulation efficiency (EE) of ONC201 were calculated using the following formulae:

LC (%) = (mass of loaded ONC201/mass of MHM) × 100

EE (%) = (mass of loaded ONC201/mass of fed ONC201) × 100

Evaluation of $\cdot O_2^-$ and $^1O_2$ Generation by US Activation. MHMO NPs (300 μg mL^{-1}) and DPBF (20 μg mL^{-1}) were dispersed in 3.0 mL of PBS (pH = 7.4). Following US exposure (40 kHz; 3.0 W cm^{-2}) for various durations, the absorbance of DPBF at 416 nm was measured to evaluate the generation rate of $\cdot O_2^-$ and $^1O_2$.

Evaluation of ·OH Generation by US Activation. MHMO NPs (300 μg mL^{-1}) and TMB solution (100 μL; 2 mg mL^{-1} in DMSO) were dispersed in 3.0 mL of PBS. Following various durations of US irradiation (40 kHz; 3.0 W cm^{-2}), the absorbance at 652 nm was measured to assess the generation rate of ·OH.

Electrochemistry Measurements. Electrochemical characterization of the MHMO NPs was performed using an electrochemical analysis instrument (CHI660E, Shanghai, China) in a Na_2SO_4 electrolyte solution (0.5 M). The instrument consisted of a three-electrode system consisting of a working electrode, a reference electrode, and an auxiliary plate (platinum plate). The working electrode was constructed by depositing 20 μL of a suspension prepared from a mixture of 10 mg NR and 2 mL 1mM Nafion-ethanol, onto an FTO glass substrate.

The Redox Potential of $H_2O/\cdot OH$. Following a previous report, it is known that $\cdot OH + e^- \rightarrow H_2O$ ($\triangle G^0$ = -244.9 kJ mol^{-1}); therefore:

$$E^0 = \frac{-\triangle G^0}{nF} = \frac{-(-244.9 \times 10^3)}{96485.3415} = 2.538\ \mathrm{V} \quad (1)$$

where n denotes the number of electrons transferred during the reaction. If it is assumed that $E^{0'} + 0.059 \times \log([\cdot OH]) = E^0$, then E_H can be calculated as follows:

$$E_H = E^{0'} - 0.059 \times \log\left(\frac{1}{[\cdot \mathrm{OH}][\mathrm{H}^+]}\right) = E^0 - 0.059 \times \mathrm{pH} = 2.10\ \mathrm{V} \quad (2)$$

Where the pH of PBS is 7.4. The E_H for ·OH generation is approximately 2.10 V at pH 7.4 concerning NHE.

Cellular Culture. The mouse colon cancer cell line CT26, embryonic fibroblast cell line 3T3, and mouse macrophages RAW264.7 were acquired from the Shanghai Cell Bank of the Chinese Academy of Sciences. Cells were cultured in either RPMI 1640 or DMEM supplemented with 10% fetal bovine serum and 1% penicillin-streptomycin at 37 °C within a humidified incubator with a 5% CO_2 atmosphere.

***In Vitro* Cellular Uptake of MHMO.** CT26 cells cultured in 6-well plates were incubated with MHMO-FITC for varying durations (0, 0.5, 2, and 4 h), followed by cell harvesting and flow cytometry analysis.

***In Vitro* Cell Viability Assay.** CT26 and 3T3 cells were plated separately in 96-well plates at a density of 5 × 10^3 cells/well and incubated overnight. The cells were divided into six groups: (1) PBS, (2) MHM, (3) MHMO, (4) US, (5) MHM + US, and (6) MHMO + US, and treated with varying drug concentrations for 40 min, followed by the addition of 20 μL MTT per well and incubation for 4 h. The absorbance was determined using a microplate reader. For rescue experiments, CT26 cells were pretreated with 20 μM z-VAD-fmk or 50 μM Nec-1 for 30 min, and the subsequent steps were performed as mentioned above**.** The Calcein/PI Cell Viability/Cytotoxicity Assay Kit was used for the visual detection of cell viability. CT26 cells were seeded in 6-well plates overnight, and drug treatments were performed as described above. Calcein AM and PI solutions were diluted 1:1000 in the buffer and co-incubated with the cells for 20 min. After washing thrice with PBS, cellular imaging was performed using a confocal laser scanning microscope (CLSM).

***In Vitro* Detection of Intracellular ROS.** Following a previously described method, CT26 cells were loaded with a DCFH-DA probe and incubated for 30 min. After three washes with PBS, the cells were mounted with an antifade mounting medium containing DAPI and observed under a CLSM.

***In Vitro* Calcium Influx Assay.** Following treatment, CT26 cells were co-incubated with 0.5 μM Fluo-4 AM for 30 min for probe loading. After three washes with PBS, fluorescence was detected using CLSM.

***In Vitro* Detection of Cell Apoptosis.** CT26 cells were plated in 6-well plates at a density of 3 × 10^5 cells/well. Following treatment as described before, cells were collected and prepared as a single-cell suspension. Following the same treatment, the cells were assessed for changes in mitochondrial membrane potential using a JC-1 Assay Kit. After 30-min of co-incubation, flow cytometric analysis was performed on the mitochondrial monomers (excitation = 490 nm, emission = 529 nm).

Western Blot. Following the grouped treatment, the cells were collected, and total protein was extracted. Protein separation was performed using 10%–12.5% SDS-PAGE, followed by transfer to a PVDF membrane. The membrane was blocked with a 5% bovine serum albumin (BSA) solution at ambient temperature for 1 h to prevent non-specific interactions.

Subsequently, the membrane was incubated with the respective primary antibodies p-RIPK1 (CST, Cat#53286S), RIPK1 (CST, Cat#3493), p-RIPK3 (Abcam, Cat#ab62344), RIPK3 (Abcam, Cat#ab195117), p-MLKL (CST, Cat#37333), MLKL (CST, Cat#37705S), Bax (Wanleibio, Cat#WL01637), Bcl2 (Wanleibio, Cat#WL01556), DR5 (Bioss, Cat#bs-1696R), and HMGB1 (abcam, Cat#ab18256) at 4 °C overnight. Following this, the membrane was washed thrice with TBST (each wash lasting 10 min), followed by a 1-hour incubation at ambient temperature with secondary antibodies. Visualization of the target protein was performed using an enhanced chemiluminescence (ECL) Western Blotting Substrate (Solarbio, PE0010).

***In vitro* cell Bio-Transmission Electron Microscopy (Bio-TEM).** CT26 cells were seeded at a density of 2×10^5 in 6-well plates and cultured overnight. After treatment with MHMO for 0, 0.5, 2, and 4 h, the cells were exposed to ultrasound at 40 kHz and 3.0 W cm^{-2} for 5 min. Afterwards, the cells were washed three times with PBS, enzymatically detached using trypsin, and centrifuged at 3,000 rpm for 15 min. After removing the supernatant, the cells were gently fixed overnight in 2.5% glutaraldehyde. Bio-TEM was used to investigate the phagocytic uptake of materials and cell death.

***In Vitro* Detection of Immunogenic Cell Death (ICD).** CT26 cells were cultured in 6-well plates and subjected to various drug treatments, as previously described. Following treatment, the cells were stabilized using 4% paraformaldehyde (PFA) for 30 min and washed thrice with PBS. Permeabilization was performed using 0.3% Triton X-100 followed by three additional PBS washes. Finally, cells were incubated at 4 °C with primary antibodies specific to HMGB1 and calreticulin (CRT) (Abcam, Cat#ab92516) after blocking with 5% bovine serum albumin (BSA) at room temperature for 1 h. On the second day, cells were treated with secondary antibodies for 1 h at room temperature and subsequently visualized using CLSM.

***In Vitro* DC Maturation Study.** Bone marrow-derived dendritic cells (BMDCs) were isolated from 6-week-old BALB/c mice and stimulated with IL-4 (10 ng ml^{-1}) and GM-CSF (20 ng ml^{-1}) for 5 days. Subsequently, the isolated DCs were co-cultured with treated CT26 cells under distinct experimental conditions for 24 h. Following co-culture, DCs were collected, stained with fluorochrome-conjugated antibodies targeting CD11c, CD80, and CD86, and analyzed using flow cytometry. Data processing and statistical assessment were performed using the FlowJo software. The levels of IL-6 and IL-12p70 released by mature DCs into the culture medium were assessed using ELISA.

MHMO + US Treatment Induces Immune Activation in Orthotopic and Bilateral Mouse Models *in Vivo*. CT26 cells (5×10^5 cells/mouse) were subcutaneously injected into the right dorsal region of the mice. One week post-inoculation, the mice were randomly divided into seven distinct groups: (1) PBS; (2) ONC201; (3) MHM; (4) MHMO; (5) US irradiation for 3 min (40 kHz; 3.0 W cm^{-2}; 3 min; 50% duty cycle); (6) MHM + US; and (7) MHMO + US. The Ca^{2+} dose used in this experiment was 47.5 μmol, while the Mg^{2+} dose was 2.5 μmol.

Mice were exposed to US 6 h after tail vein injection, and the treatment was repeated every 3 days until day 20. Following this period, spleens were collected and processed into single-cell suspensions for subsequent analyses. The proportions of mature DCs ($CD11c^+$, $CD80^+$, and $CD86^+$) and $CD8^+$ T cells ($CD45^+$, $CD3^+$, and $CD8^+$) were assessed using flow cytometry and analyzed using FlowJo software. To establish a murine model of distant metastasis, six-week-old BALB/c mice were subcutaneously injected with CT26 cells (5×10^5 cells/mouse) in the right flank. One week later, a subsequent injection of CT26 cells (2.5×10^5 cells/mouse) was administered subcutaneously on the left flank. Following the second inoculation, mice were randomized into seven groups and subjected to the aforementioned treatment regimen. The tumor volumes ($V = \text{length} \times \text{width}^2/2$) and body weights of the mice were monitored at 3-day intervals throughout the study period. At the end of day 21, the mice were euthanized, and tumor tissues were collected to analyze immune cell infiltration within the tumor microenvironment. Mouse tumor tissues were digested with collagenase to generate single-cell suspensions. Subsequently, flow cytometry analysis was performed to characterize the following immune cell populations: $CD8^+$ T cells ($CD45^+$, $CD3^+$, and $CD8^+$), cytotoxic T lymphocytes ($CD3^+$, $CD8^+$, IFN-γ^+, and CTLs), M1 macrophages ($CD11b^+$, $F4/80^+$, and $CD86^+$), and M2 macrophages (CD11b+, $F4/80^+$, and $CD206^+$). The remaining mouse tumor tissues were fixed with PFA, dehydrated, and subsequently processed for frozen sectioning and immunostaining using CD8, iNOS, and CD206, enabling the direct visualization of immune cell infiltration within the tumor microenvironment. Concurrently, serum samples were harvested and analyzed for the detection of the TNF-α and IFN-γ cytokines using ELISA techniques.

Efficacy of MHMO + US in Preventing Tumor Recurrence and Inhibiting Lung Metastasis. A tumor recurrence study was conducted using a CT26 subcutaneous tumor-bearing mouse model. Following the implementation of the different treatment regimens, the tumor masses were surgically removed. After a 20-day interval, an experimental lung metastasis model was established by injecting mice with 5×10^5 CT26-Luc cells via the tail vein. On day 14 after treatment initiation, fluorescence imaging was employed to quantify lung metastasis in

mice, followed by dissection and histological examination of the lung tissues via H&E staining. Spleens were extracted from the mice, processed into single-cell suspensions, and subjected to flow cytometric evaluation of effector memory T cells ($CD3^+$, $CD8^+$, $CD44^+$, $CD62L^-$, and Tem) and central memory T cells ($CD3^+$, $CD8^+$, $CD44^+$, $CD62L^+$, and Tcm).

Statistical Analysis. GraphPad Prism 8.0, OriginPro 9.0, and FlowJo v10.8 were utilized for graphical representations and statistical analyses. Data were subjected to statistical analysis using a two-tailed Student's *t*-test or two-way ANOVA, as applicable. The results are presented as the mean ± SD. Statistical significance was set at $p < 0.05$. n.s. indicates no significance; asterisks represent significant differences (*$p < 0.05$, **$p < 0.01$, ***$p < 0.001$, and ****$p < 0.0001$).

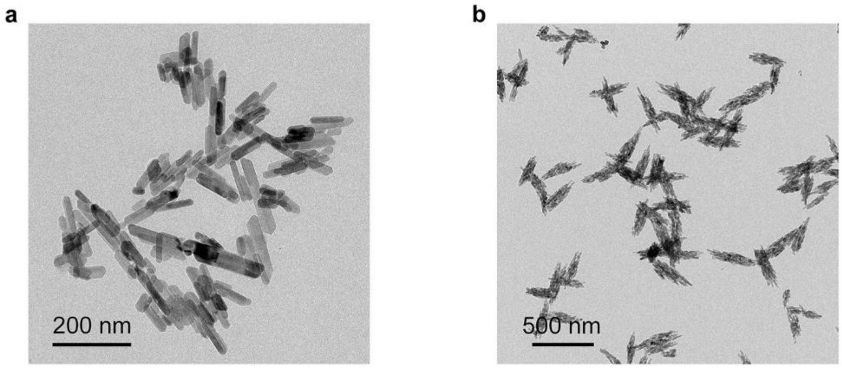

Figure S1. TEM image of Mg-HAP NPs with Ca: Mg ratios of a) 3.9:0.1 and b) 3.7:0.3

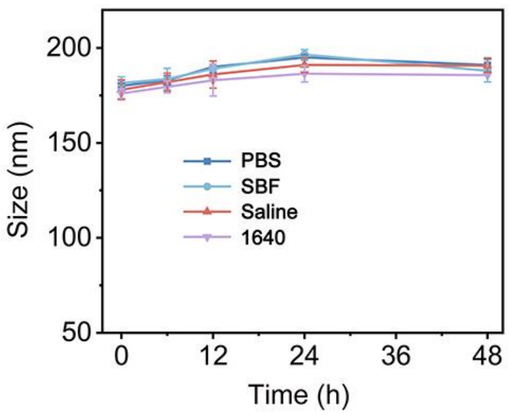

Figure S2. Dynamic light scattering (DLS) size distribution profiles of MHMO in different media across various time points.

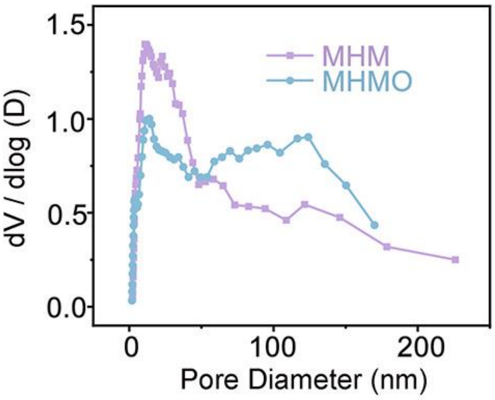

Figure S3. The corresponding pore sizes of MHM and MHMO NPs.

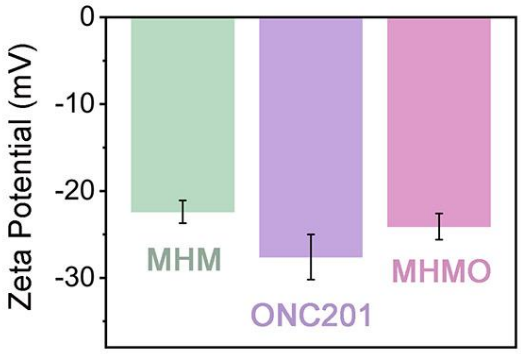

Figure S4. Zeta potentials of the synthesized materials at different steps.

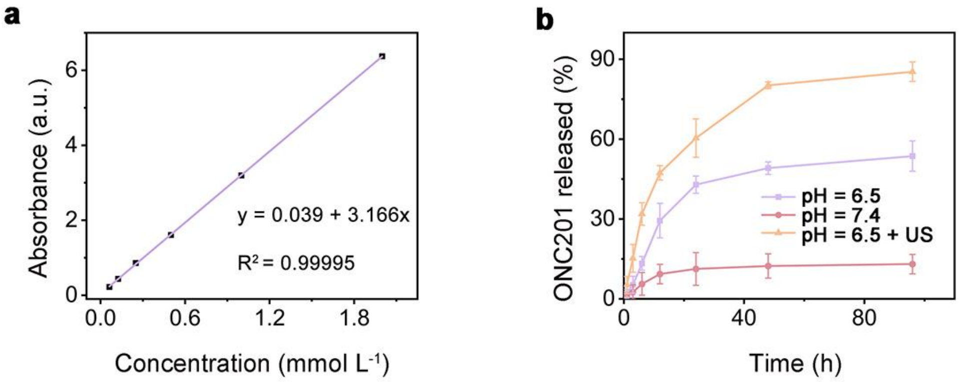

Figure S5. a) The standard concentration-dependence curve of ONC201 at different absorbances under a wavelength of 388 nm. b) Cumulative release curves of ONC201 from MHMO NPs at pH = 6.5, pH = 7.4, and pH = 6.5 + US.

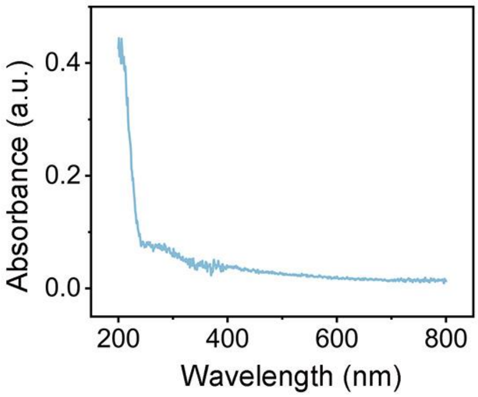

Figure S6. The UV-*vis* diffuse reflectance spectrum of MHMO NPs.

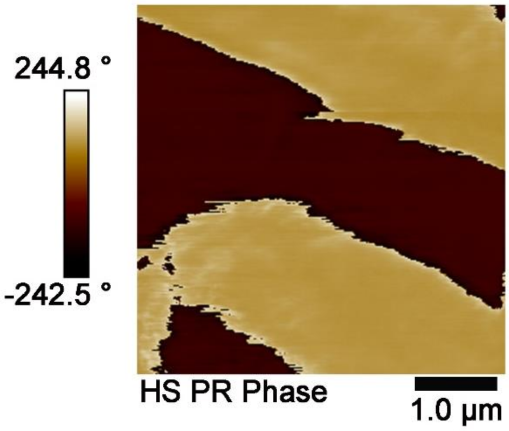

Figure S7. The phase image of MHMO NPs.

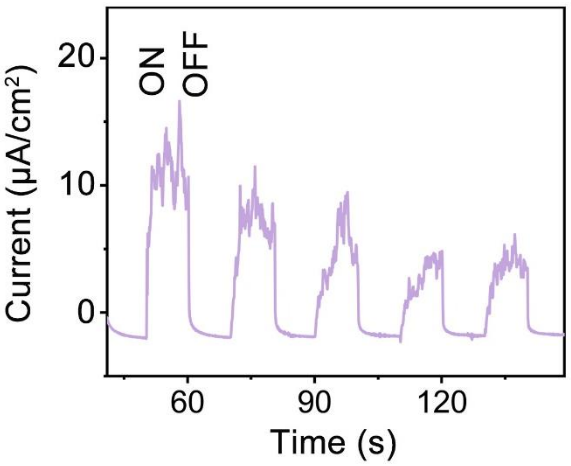

Figure S8. The piezocurrent of MHMO NPs.

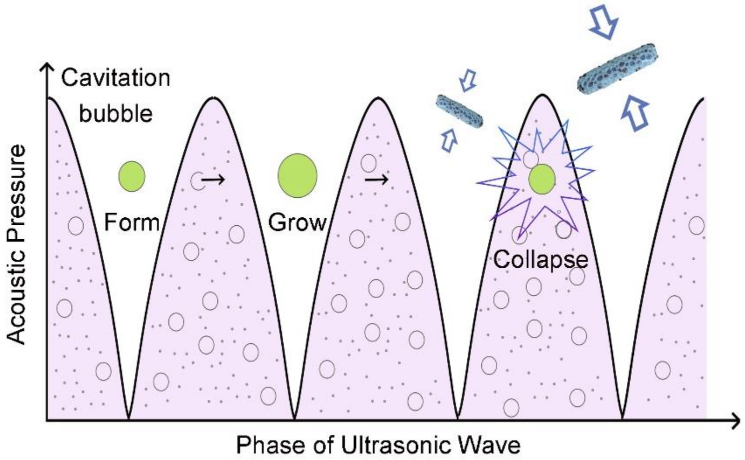

Figure S9. The propagation of the acoustic waves instigates cavitation bubble formation, growth, and collapse within the fluid. This bubble implosion results in pressure fluctuations, promoting the internal polarization of the nanoribbons.

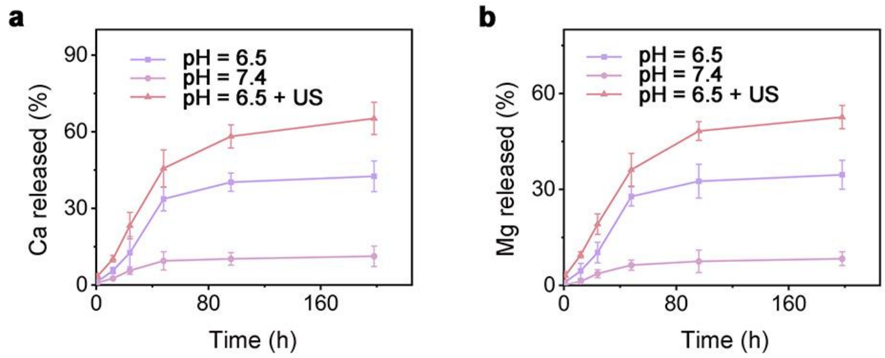

Figure S10. Cumulative release of a) Ca and b) Mg from MHMO at pH = 6.5, pH = 7.4, and pH = 6.5 + US.

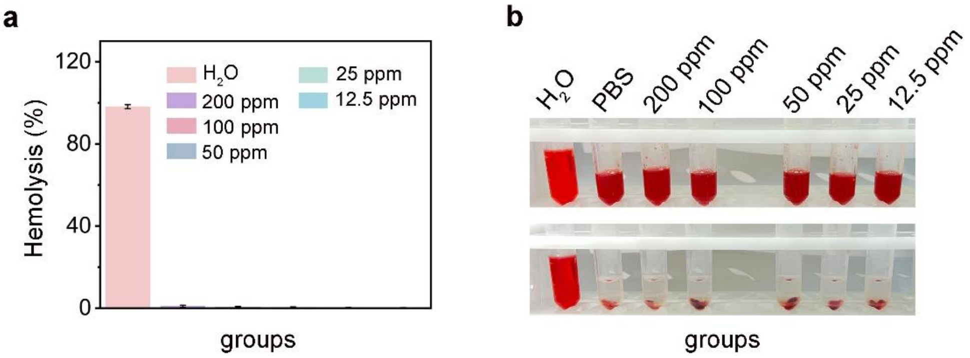

Figure S11. *In vitro* hemolysis test. a) Hemolysis analysis was performed on MHMO at concentrations of 12.5, 25, 50, 100, and 200 ppm, dispersed in PBS solution. b) Images of hemolysis experiments.

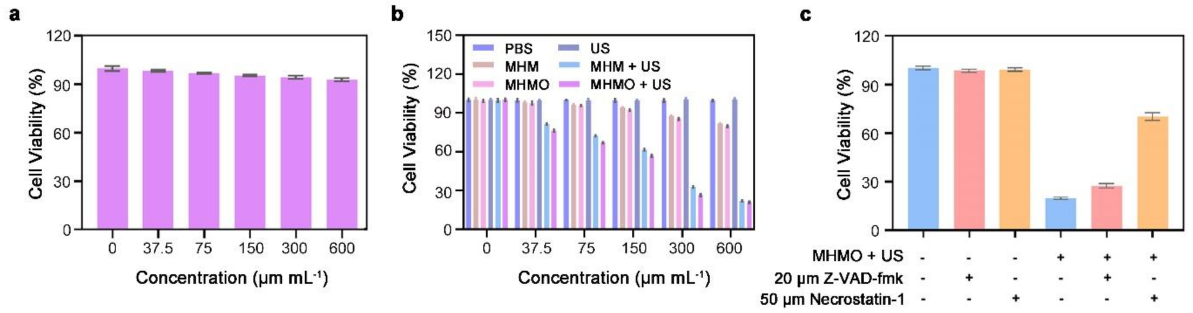

Figure S12. a) The viability of 3T3 cells following a 24-h exposure to various concentrations of MHMO NPs. b) Cytotoxicity profiles of CT26 cells following various treatments. c) CT26 cells were pretreated with varying concentrations of z-VAD-fmk or Nec-1 for 30 min and then co-cultured with MHMO + US for 24 h, following which the cell viability was measured using an MTT assay.

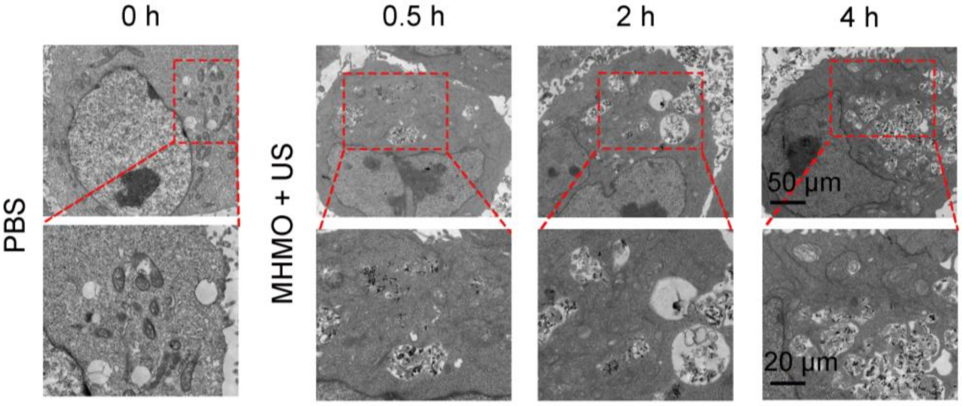

Figure S13. Biological TEM was used to evaluate the cellular phagocytosis of the materials after 0, 0.5, 2, and 4 h. The scale bar indicates 50 or 20 μm.

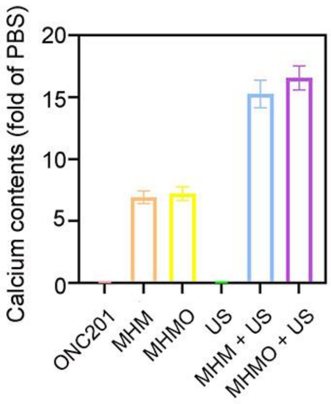

Figure S14. The calcium concentrations in CT26 cells, compared with the PBS group.

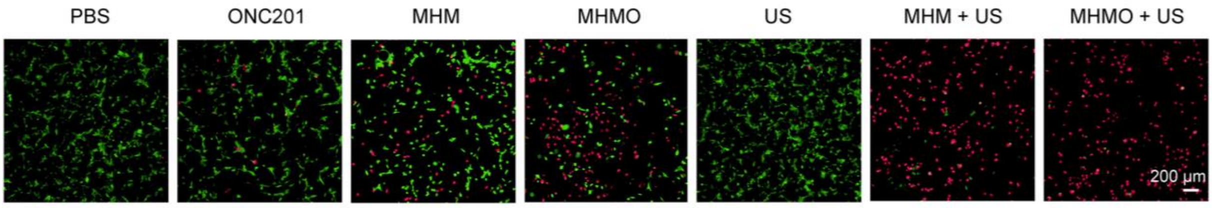

Figure S15. CT26 cells underwent Calcein-AM/PI double staining following exposure to various treatments. The scale bar represents 200 μm.

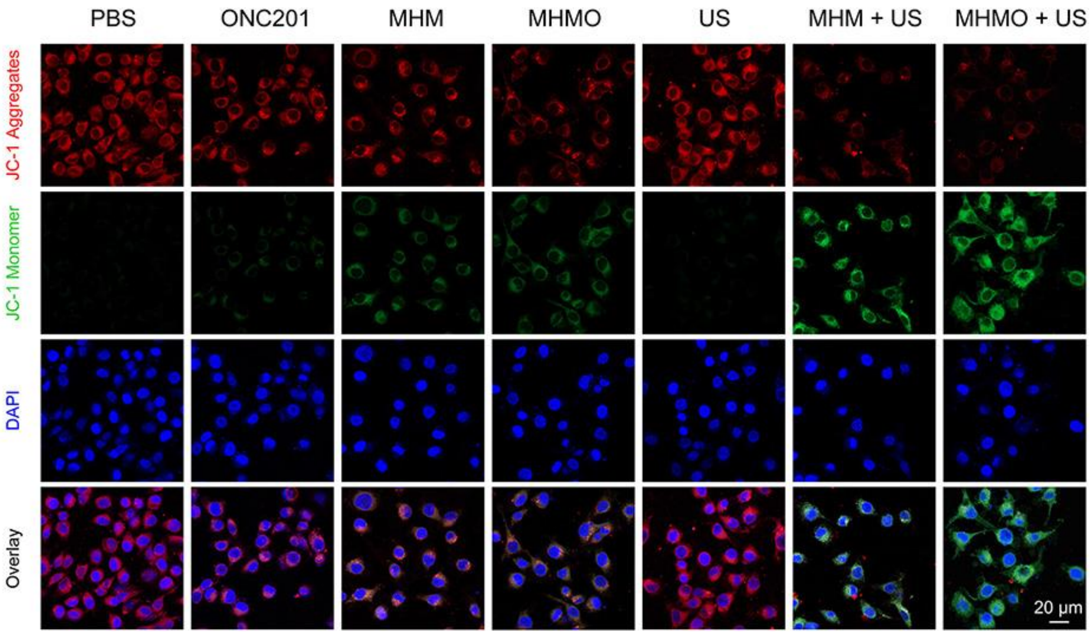

Figure S16. The JC-1 assay was performed on CT26 cells to evaluate changes in mitochondrial membrane potential using immunofluorescence following various treatments.

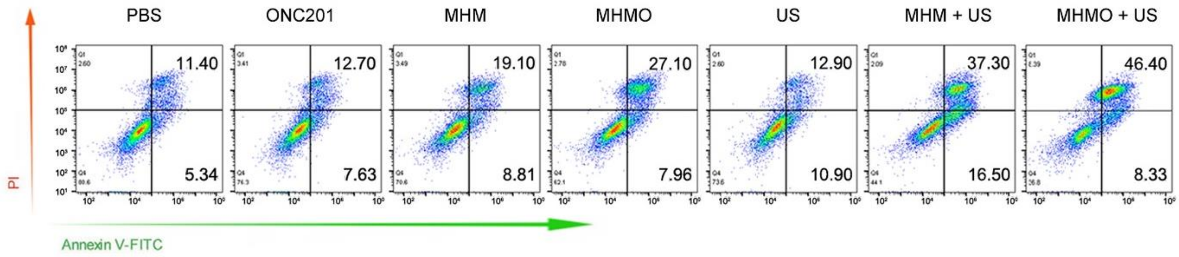

Figure S17. Flow cytometry was employed to measure apoptosis in CT26 cells following various treatments.

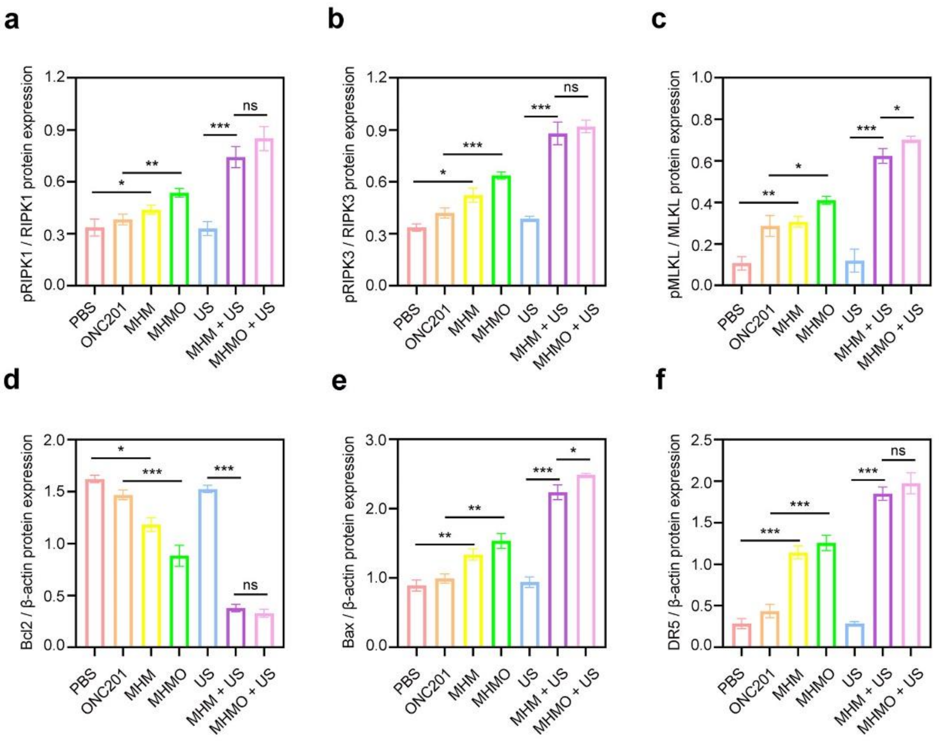

Figure S18. Western blot assays were performed to determine the levels of necroptosis, apoptosis, and DR5. a-c) Quantification of p-RIPK1, RIPK1, p-RIPK3, RIPK3, p-MLKL, and MLKL. d, e) Quantification of Bax and Bcl2. f) Quantification of DR5.

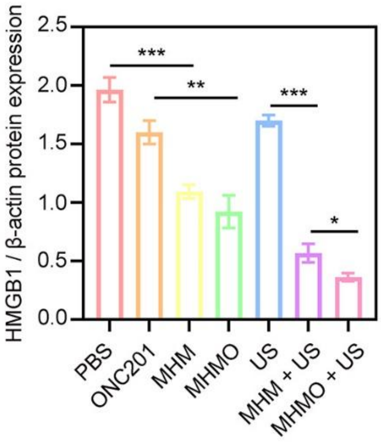

Figure S19. Quantitative analysis of HMGB1 by western blotting.

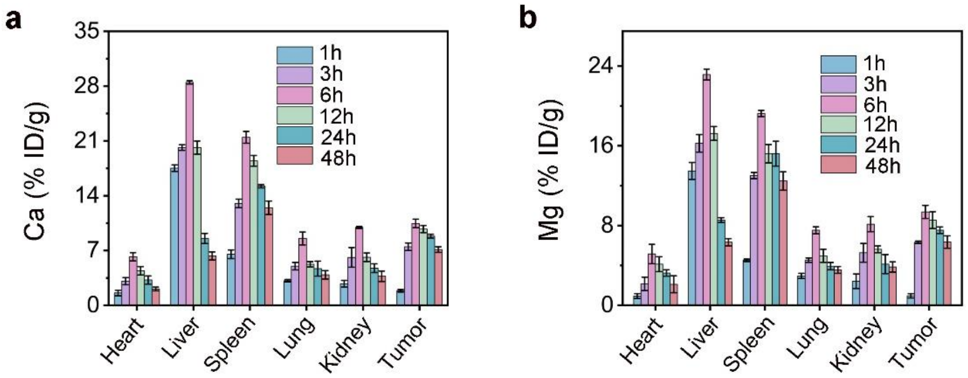

Figure S20. Biodistribution of a) Ca and b) Mg in various organs and tumors of mice. Data are represented as the mean ± S.D. (n = 3).

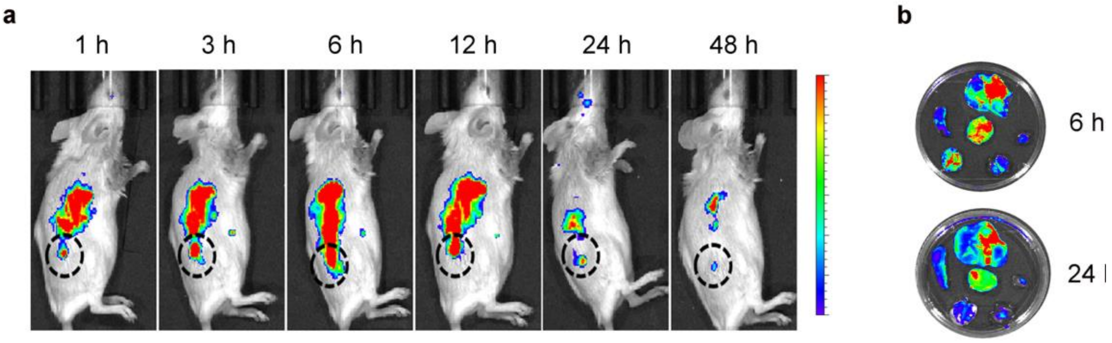

Figure S21. Fluorescence images a) *in vivo* b) and *in vitro* at various time points following intravenous injection.

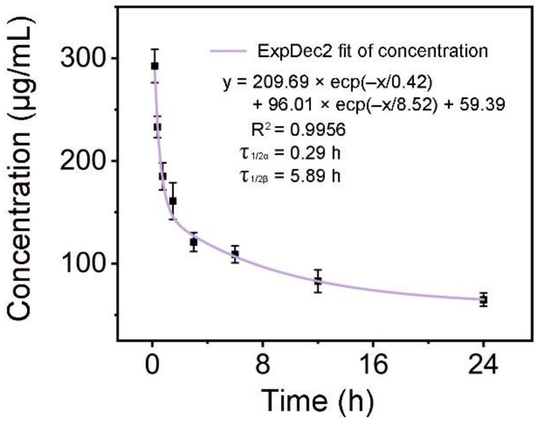

Figure S22. Blood circulation curve of mice injected with MHMO through the tail vein. Data are represented as the mean ± S.D. (n = 3).

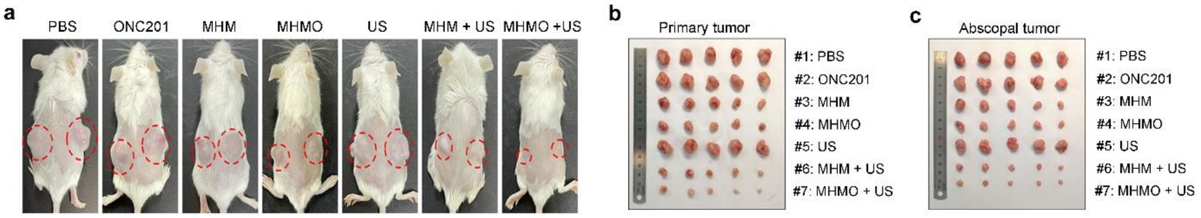

Figure S23. CT26 cells were injected into BALB/c mice (n = 5). Tumor mass was was evaluated on the 20th day post-establishment of the tumor model and commencement of treatment. a) Macroscopic images of mouse anatomy. b) Primary tumor; c) Abscopal tumor.

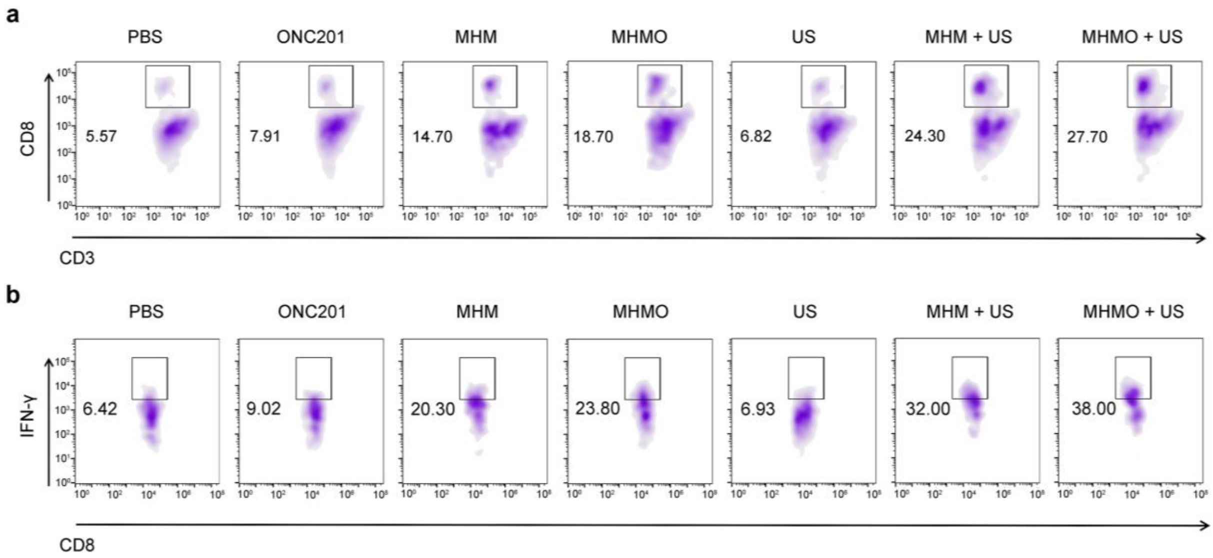

Figure S24. Post-therapy, distant tumor tissues harvested from mice were converted into cell suspensions, and subsequent staining was performed to delineate a) $CD8^+$ T cells ($CD3^+$ and $CD8^+$, gating $CD45^+$) and b) CTLs ($CD3^+$, $CD8^+$, and IFN-γ^+) via flow cytometry.

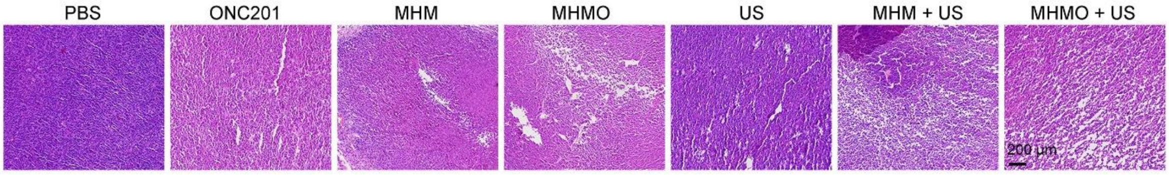

Figure S25. Representative images depicting H&E staining in tumor tissue sections across various treatment groups. The scale bar represents 200 μm.

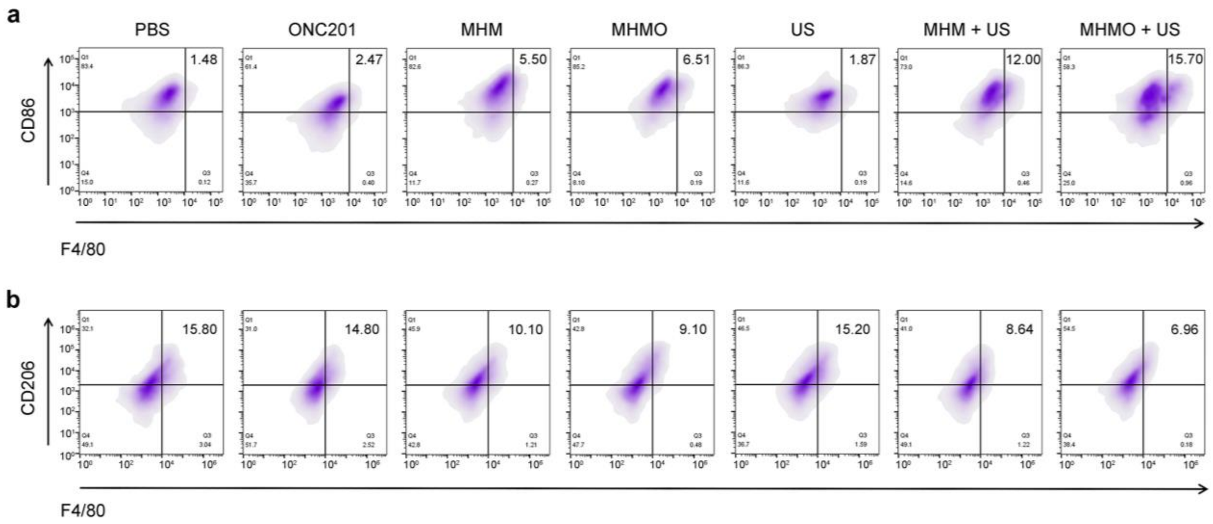

Figure S26. Flow cytometric evaluation of the infiltration ratios of a) M1-like ($CD86^+$) macrophage and b) M2-like ($CD206^+$) macrophage (gating $F4/80^+$ and $CD11b^+$) within distant tumor tissues.

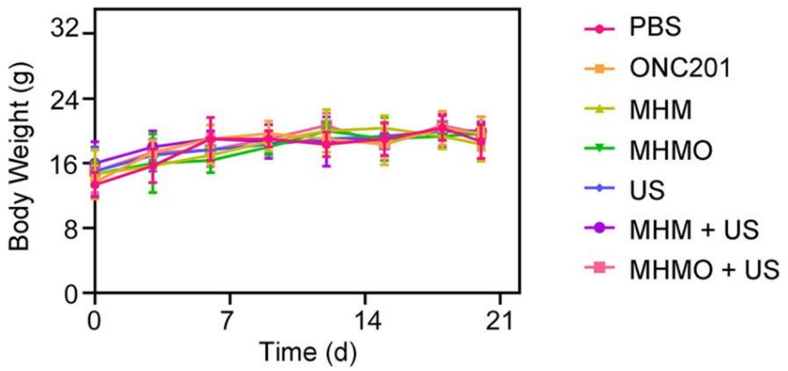

Figure S27. Changes in mice body weight following the various treatments. The data are illustrated as the mean values ± SD (n = 5).

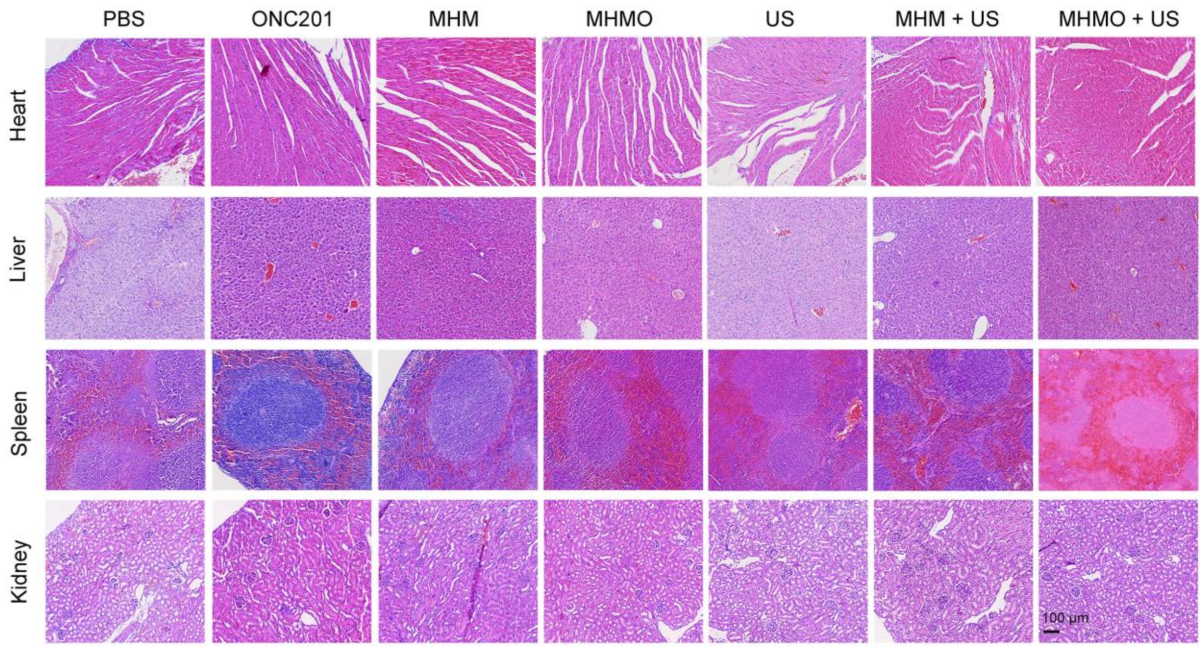

Figure S28. H&E staining images of the heart, liver, spleen, and kidney after treatments. The scale bar indicates 100 μm.

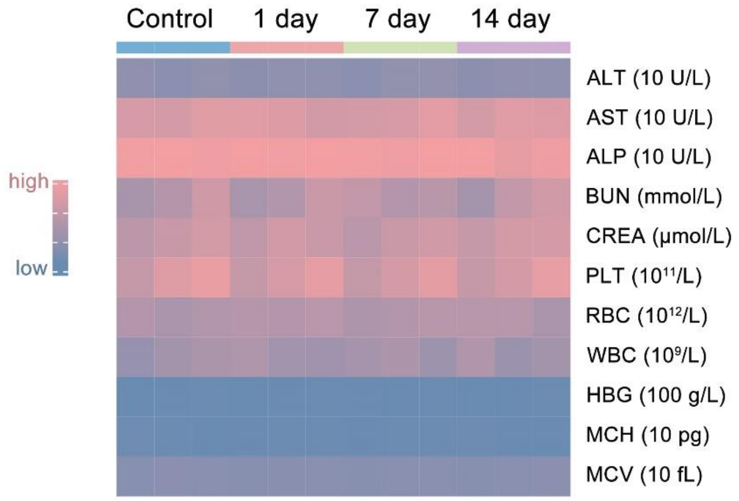

Figure S29. The blood biochemistry and hematological test results following MHMO + US treatment in mice at various time points. The blood biochemistry includes the liver function indicators alkaline phosphatase (ALP), alanine aminotransferase (ALT), aspartate aminotransferase (AST), blood urea nitrogen (BUN), and blood creatinine (CREA). The complete blood count included platelets (PLT), red blood cells (RBC), white blood cells (WBC), hemoglobin (HGB), mean corpuscular hemoglobin (MCH), and mean corpuscular volume (MCV).

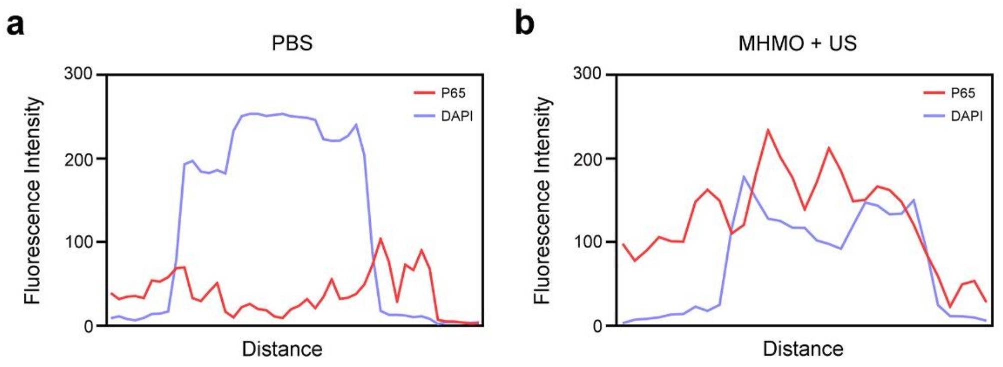

Figure S30. Fluorescence colocalization analysis showing P65 nuclear translocation following MHMO + US treatment. a) P65 nuclear translocation status in the PBS-treated group. b) P65 nuclear translocation status in the MHMO-treated group.
